# Transdifferentiation and Proliferation in Two Distinct Hemocyte Lineages in *Drosophila melanogaster* Larvae after Wasp Infection

**DOI:** 10.1371/journal.ppat.1005746

**Published:** 2016-07-14

**Authors:** Ines Anderl, Laura Vesala, Teemu O. Ihalainen, Leena-Maija Vanha-aho, István Andó, Mika Rämet, Dan Hultmark

**Affiliations:** 1 Institute of Biosciences and Medical Technology, BioMediTech, University of Tampere, Tampere, Finland; 2 Department of Molecular Biology, Umeå University, Umeå, Sweden; 3 Institute of Genetics Biological Research Centre of the Hungarian Academy of Sciences, Szeged, Hungary; 4 PEDEGO Research Unit, and Medical Research Center Oulu, University of Oulu and Oulu University Hospital, Oulu, Finland; Stanford University, UNITED STATES

## Abstract

Cellular immune responses require the generation and recruitment of diverse blood cell types that recognize and kill pathogens. In *Drosophila melanogaster* larvae, immune-inducible lamellocytes participate in recognizing and killing parasitoid wasp eggs. However, the sequence of events required for lamellocyte generation remains controversial. To study the cellular immune system, we developed a flow cytometry approach using *in vivo* reporters for lamellocytes as well as for plasmatocytes, the main hemocyte type in healthy larvae. We found that two different blood cell lineages, the plasmatocyte and lamellocyte lineages, contribute to the generation of lamellocytes in a demand-adapted hematopoietic process. Plasmatocytes transdifferentiate into lamellocyte-like cells *in situ* directly on the wasp egg. In parallel, a novel population of infection-induced cells, which we named lamelloblasts, appears in the circulation. Lamelloblasts proliferate vigorously and develop into the major class of circulating lamellocytes. Our data indicate that lamellocyte differentiation upon wasp parasitism is a plastic and dynamic process. Flow cytometry with *in vivo* hemocyte reporters can be used to study this phenomenon in detail.

## Author Summary

Hematopoiesis is regulated by a conserved set of signaling pathways in flies and men. While the implication of multiplex flow cytometry has led to the discovery of a plethora of new human blood cell types, it has not routinely been used to study fly hematopoiesis. We present here the application of flow cytometry with *in vivo* blood cell reporter constructs to study infection-induced hematopoiesis in blood samples from single fly larvae. We put forward a new perspective on *Drosophila* hematopoiesis, based on our discovery of new blood cell types. We show a switch from steady-state to demand-adapted hematopoiesis in fly larvae, which corresponds well to the described demand-adapted hematopoiesis after infection and inflammation in humans. Our results provide new insights into the dynamics of infection-induced hematopoiesis in fly larvae and increase the versatility of the fly as a model system to study the cellular innate immune system.

## Introduction

Blood cells are the central players in the cellular immune response, and evolutionarily conserved signaling pathways control their hematopoiesis. Three main types of blood cells or hemocytes have been described for *Drosophila melanogaster*; plasmatocytes, crystal cells, and lamellocytes. Plasmatocytes, the main hemocyte type in healthy larvae, are professional phagocytes, and they are functionally similar to mammalian monocytes, macrophages, and neutrophils. Lamellocytes are formed in response to wasp infection, when they are needed for the encapsulation and killing of parasitoids. Finally, crystal cells, are required for the melanization of wounds. Together with lamellocytes, crystal cells probably also contribute to the melanization of capsules, a presumed effector mechanism of the immune defense [1–5].

A variety of parasitoid hymenopteran wasp species, including the genus *Leptopilina* [6], deposit their eggs in the hemocoel of fly larvae. This triggers a melanotic encapsulation reaction that comprises a fixed sequence of events. After the egg is injected, a thin electron-dense layer of unknown material is deposited on the chorion. Then plasmatocytes attach to and spread on the egg. Several layers of lamellocytes encapsulate the egg, the capsule is sealed by septate junctions between the cells, and finally melanin is deposited by the action of the enzyme phenol oxidase [7,8]. Both crystal cells and lamellocytes participate in the melanization reaction [5,9]. Parasitoid wasp species, in turn, deploy several virulence strategies, which incapacitate the host´s cellular immune system in different ways and visibly affect hemocytes [10].

*Drosophila* larval hemocytes originate from two embryonic sources; the procephalic and the cardiogenic mesoderm anlagen [11]. The cardiogenic anlage gives rise to two rows of hematopoietic organs, called lymph glands, which are situated on each side of the dorsal vessel. The paired primary lobes of the lymph glands consist of a medullary zone with progenitor cells, a cortical zone with differentiated hemocytes, and a posterior signaling center that supervises the maintenance and differentiation of progenitor cells [12–14]. Prior to pupariation, the lymph glands disintegrate and release mature hemocytes. In response to wasp infection the primary lobes can disintegrate earlier and release differentiated plasmatocytes and lamellocytes [15,16]. The hemocytes of procephalic origin give rise to the peripheral hemocyte population, which is distinct from the lymph glands. Peripheral hemocytes colonize a second hematopoietic compartment of sessile hemocyte islets, which are arranged in segments under the skin [15,17,18]. These hemocytes proliferate in contact with peripheral neurons [19] and alternate between sessile positions in the islets and circulation in the open body cavity of larvae [4,19]. In healthy larvae, all hemocytes are of procephalic origin until the onset of metamorphosis, when hemocytes are released from the lymph glands. Procephalic hemocytes also persist into adulthood [4]. Although research during the past 15 years has highlighted the lymph glands as the source of lamellocytes, Rizki suggested already in 1957 that peripheral plasmatocytes give rise to lamellocytes [20]. This idea was more recently corroborated by lineage tracing in three studies [21–23]. After a wasp attack, a main fraction of the hemocytes participating in the encapsulation reaction originates from the peripheral population [18,21]. Nevertheless, the origin of lamellocytes remains ambiguous, and the dynamics of the cellular immune system in the encapsulation reaction is unclear.

Initially, hemocytes were classified by their morphology [20]. The development of the enhancer trap system in *Drosophila* enabled the production of the first generation of genetic hemocyte markers [24,25]. Later, hemocyte-specific antibodies provided pan-hemocyte antibodies [26] as well as specific antibodies for the different hemocyte classes [27,28]. These antibodies were also instrumental in the discovery of new hemocyte-specific proteins. Hemocyte-specific GAL4 constructs and fluorescent enhancer-reporter fusions further diversified the genetic toolbox, allowing the observation of hemocytes or specific hemocyte subclasses *in vivo* [17,29–33]. These, and other markers used to study embryonic hemocytes and lymph glands, are reviewed in [34]. Despite these advances, peripheral hemocytes are mainly counted with hemocytometers, which is labor-intensive and error-prone. So far, the use of flow cytometry in the differential cell counting and sorting of *Drosophila* hemocytes has been minimal.

Here we present a combined approach of flow cytometry and microscopy to investigate the dynamics of hematopoiesis after a wasp infection. We took advantage of the previously developed enhancer-reporter constructs *eater*-*GFP* (here called *eaterGFP*) [30], which is specific for plasmatocytes, and *MSNF9MO*-*mCherry* (*msnCherry*), which is specific for lamellocytes [32]. We chose three species of the parasitoid wasp genus *Leptopilina*, each with different well-established effects on the immune response of *Drosophila* larvae, to better understand the origin of lamellocytes and the dynamics of the hemocyte compartments during the encapsulation reaction. We show that flow cytometry, combined with fluorescent enhancer-reporter constructs, is an effective way to distinguish different hemocyte classes. A wasp infection induces several novel hemocyte classes that belong to two major lineages, the plasmatocyte and the lamellocyte lineage. These lineages give rise to two types of lamellocytes and activated plasmatocytes in a demand-adapted response.

## Results

### Flow cytometry detects six different blood cell types

To simultaneously monitor plasmatocytes and lamellocytes, we recombined X chromosome insertions of the *msnCherry* and *eaterGFP* reporters to generate a dual reporter chromosome, here referred to as *Me*. We will refer to the corresponding expressed phenotypes as mCherry and GFP, respectively. We chose three wasp species of the genus *Leptopilina* to study the encapsulation and killing response in *Me*/*w* ^*1118*^ *iso* (*Me*/*w*) heterozygous larvae. *L*. *boulardi* is a specialist for *D*. *melanogaster* that kills approximately half of the infected host larvae after interfering with hemocyte spreading [35,36] and the melanization reaction [37,38]. *L*. *heterotoma* is a generalist species that kills almost all infected larvae [39] by lysing lamellocytes [40,41]. *D*. *melanogaster* is not a host species for *L*. *clavipes*, and eggs of this species are efficiently killed by the larval immune system [6]. We assumed that the different life styles of these wasp species would be reflected in differences in the immune responses of *Drosophila* larvae. As expected, the eggs and larvae of the three wasp species were melanized and killed with different efficiencies (S1A Fig). *L*. *boulardi* eggs were melanized at a low rate (examples are shown in S1B–S1B”“Fig). Most of the wasp larvae hatched 30–32 h after infection. Thereafter the *Drosophila* larval cellular immune system mounted a melanotic encapsulation response and killed about 40% of the wasp larvae (examples of living or killed wasp larvae are shown in S1C and S1C” Fig). *L*. *clavipes* eggs were more readily encapsulated and living wasp larvae of that species were rarely found in the hemocoel. In contrast, a melanotic encapsulation response never developed against *L*. *heterotoma* eggs or larvae.

**Fig 1:**
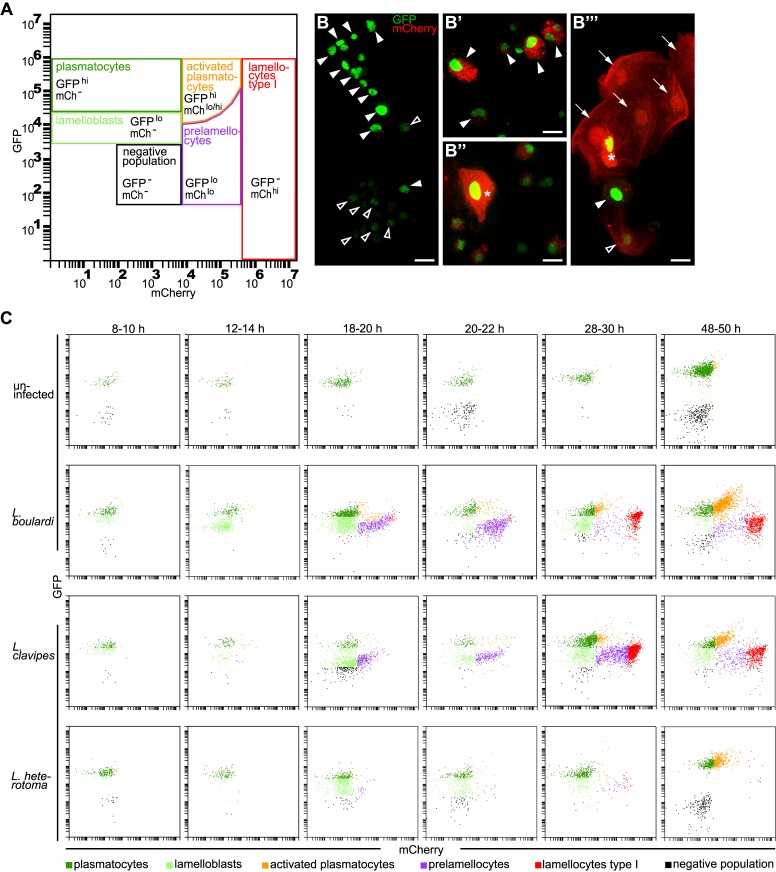
Six different hemocyte classes. (A) Hemocyte gates. Plasmatocytes (GFP^hi^mCh^-^) and lamelloblasts (GFP^lo^mCh^-^) expressed only GFP. Activated plasmatocytes (GFP^hi^mCh^lo^) were equal in size to plasmatocytes and had varying amounts of mCherry-positive punctae in their cytoplasm. Similar to activated plasmatocytes, type II lamellocytes expressed both markers (GFP^hi^ mCh^hi^). Type II lamellocytes were larger than plasmatocytes, similar in shape to lamellocytes, and with strong expression of mCherry in their cytoplasm. Prelamellocytes (GFP^lo^mCh^lo^) had low GFP expression while increasing their mCherry expression. Type I lamellocytes (GFP^-^mCh^hi^) expressed only mCherry. The negative population expressed neither GFP nor mCherry. (B-B”‘) Representative images of hemocyte types. (B) plasmatocytes (filled arrowheads) and lamelloblasts (open arrowheads). (B’) Activated plasmatocytes (filled arrowheads). (B”) Type II lamellocyte (star). (B”‘) Lamellocytes type I (arrows), prelamellocyte (open arrowhead), activated plasmatocyte (filled arrowhead), and lamellocyte type II (star). Scale bars 10 μm. (C) Flow cytometry plots at representative time points after a wasp infection. *Me/w* larvae were uninfected or infected by *L*. *boulardi*, *L*. *clavipes*, and *L*. *heterotoma*. The time points were chosen to cover major changes in the composition of the hemocyte population in the course of the time line experiment. In uninfected animals mainly plasmatocytes were present in the circulation. The cellular immune response after infection by *L*. *boulardi* and *L*. *clavipes* proceeded in a stereotypical way. At 10–12 h after infection, two plasmatocyte-like populations, plasmatocytes and lamelloblasts, were present in the circulation. At 18–20 h after infection, lamelloblasts developed into prelamellocytes. Plasmatocytes started to appear already 8–10 h after infection, and were the dominant cell type at 48–50 h. The first type I lamellocytes were seen in the circulation 20–22 h after infection. Large numbers of type I lamellocytes were in the circulation 28–30 h and 48–50 h after infection. A *L*. *heterotoma* infection induced a similar immune response until 18–20 h after infection. Then the numbers of prelamellocytes and lamellocytes type I were reduced in comparison to *L*. *boulardi* and *L*. *clavipes*-infected larvae. 48–50 h after a *L*. *heterotoma* infection, plasmatocytes and activated plasmatocytes were the dominant hemocyte types present. They were accompanied by only very few type I lamellocytes.

After we had confirmed the unequal encapsulation responses against these wasp species, we set out to characterize the dynamics of the cellular immune response with flow cytometry and microscopy. Our first approach was a time line experiment where we let *L*. *boulardi* females lay eggs in heterozygous *Me*/*w* larvae for two hours. Then we bled infected and age-matched control larvae every second hour during a time course of 50 h and analyzed hemocyte numbers and types by flow cytometry. A detailed gating strategy can be found in Fig 1A and S2 Fig. In S3 Fig, we illustrate the morphology of the different cell types after cell sorting, thereby verifying our gating strategy.

As expected, we detected only a single well-defined population of GFP-positive plasmatocytes in the hemolymph of uninfected animals by flow cytometry (Fig 1C, upper row). In contrast, a *L*. *boulardi* infection induced drastic changes producing an increased diversity of hemocyte phenotypes (Fig 1A, 1B and 1C, second row). Besides the typical plasmatocytes, a second heterogeneous population of plasmatocytes appeared. This class, which we named activated plasmatocytes, expressed high levels of GFP together with varying amounts and variable sizes of cytoplasmic mCherry-positive foci (Fig 1B’). In addition to the pre-existing plasmatocytes, we found a separate population of cells with plasmatocyte-like morphology, but with 10-fold lower GFP fluorescence (Fig 1B and 1C, second row, S4A and S4A’ Fig). We will tentatively refer to these cells as lamelloblasts because they appear to be a major source of circulating lamellocytes. Apart from differences in GFP expression levels, lamelloblasts were less granular and slightly smaller than plasmatocytes (S4C and S4C’ Fig). A fourth class gradually appeared, with increasing mCherry expression and correspondingly decreasing GFP expression. Morphologically these were intermediates between lamelloblasts and lamellocytes, and we will refer to them as prelamellocytes (Fig 1B”‘). The fifth class, the fully differentiated lamellocytes, represented the end result of this development. They were large cells that only expressed mCherry throughout the cytoplasm (Fig 1B”‘). A sixth class included large cells with homogeneous expression of mCherry throughout the entire cytoplasm, however, unlike circulating lamellocytes, they also expressed strong nuclear GFP (Fig 1B” and 1B”‘). These cells were rarely observed in circulation. To distinguish them from circulating lamellocytes (lamellocytes type I), we will refer to them as lamellocytes type II. Finally, there was also a highly variable number of cells or particles, defined as the "negative population", that did not express either of the two reporter constructs (Fig 1A). This fraction may include crystal cells and prohemocytes as well as cell fragments and other debris. Due to the lack of good markers we did not further investigate these cells.

### Dynamics of circulating hemocytes over time

**Fig 2:**
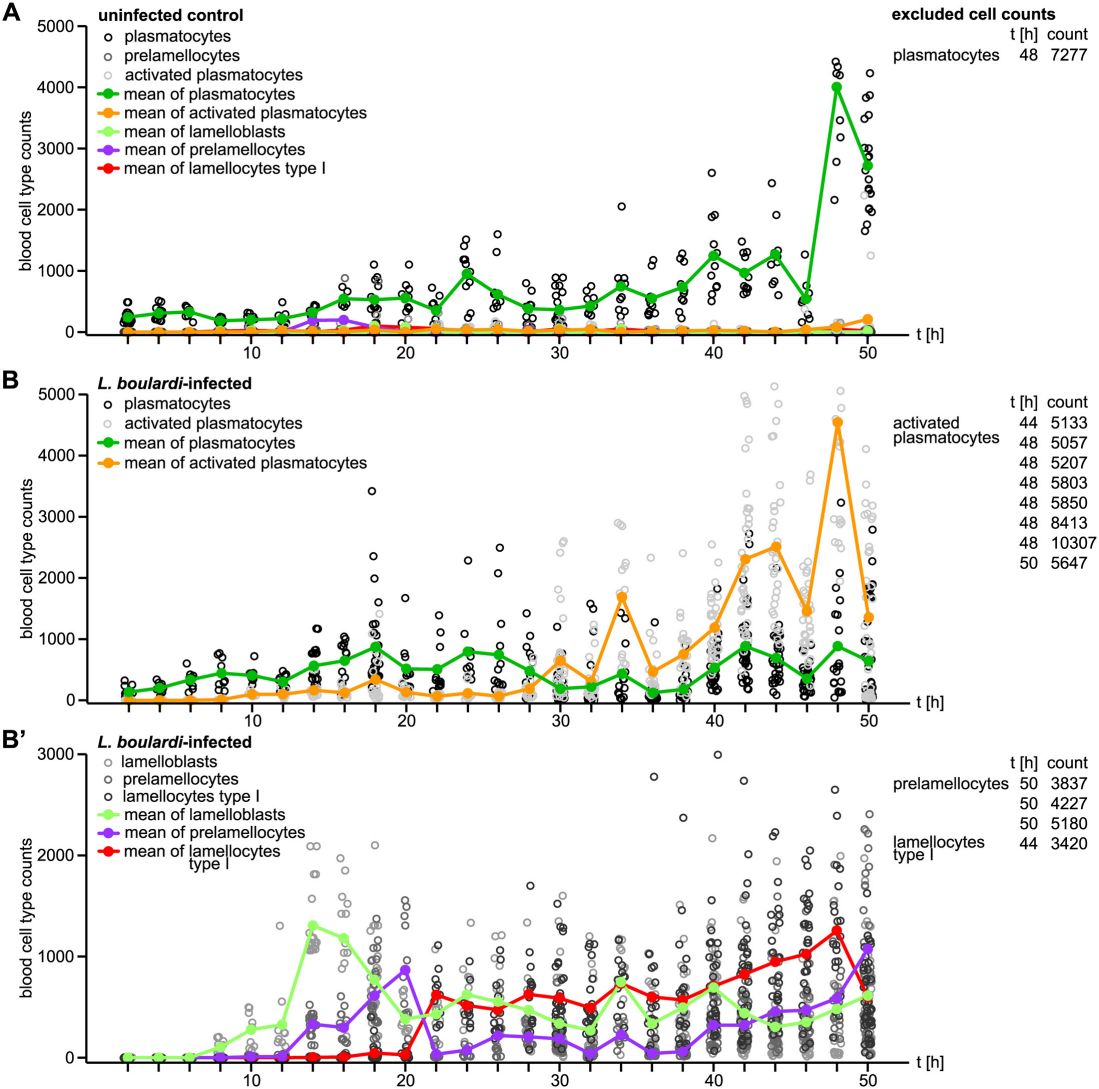
Timeline of circulating hemocyte counts of age-matched control and wasp-infected larvae. (A) Total counts of each hemocyte type of uninfected *Me/w* heterozygous larvae collected every second hour until 50 h after infection. (B) Total counts of plasmatocytes and activated plasmatocytes, as well as (B’) lamelloblasts, prelamellocytes, and type I lamellocytes collected every second hour until 50 h after infection by *L*. *boulardi*. The hemocyte population of uninfected control animals consisted mainly of plasmatocytes, whereas the hemocyte populations of infected animals followed a pattern of a demand-adapted hematopoiesis. In order to facilitate plotting, we excluded hemocyte counts of few individual larvae that clearly deviated from the rest. Cell types, time points, and cell counts are presented in tables next to each plot.

A time line experiment revealed the dynamics of the cellular immune response (Fig 2). Total cell numbers in *L*. *boulardi*-infected larvae were higher than in uninfected larvae. The increase of total cell numbers started eight to ten hours after infection (S5A and S5B Fig).

Most of the circulating blood cells in uninfected larvae were plasmatocytes. Only in late third instar larvae a few activated plasmatocytes developed. Plasmatocyte numbers increased steadily but never reached much more than 1000 cells per larva, until a sudden increase prior to pupariaton (Fig 2A).

**Fig 3:**
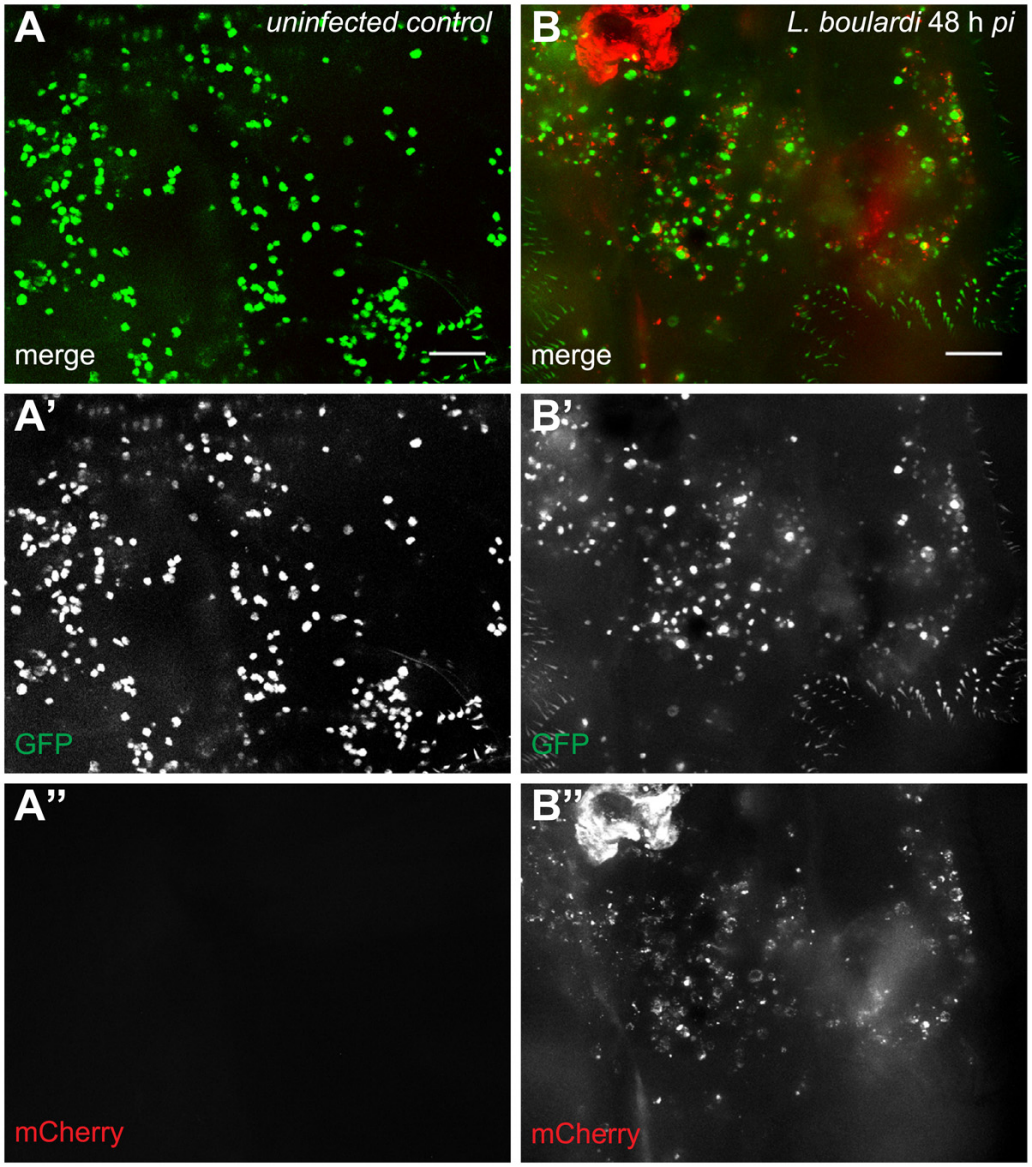
Activated plasmatocytes. Sessile hemocytes of (A-A”) uninfected *Me/w* control larvae and (B-B”) *L*. *boulardi*-infected *Me/w* larvae 48 h after infection. The images show the terminal segments of two *Drosophila* larvae. In uninfected controls, plasmatocytes were the predominant sessile cell type, whereas in infected animals activated plasmatocytes were prevalent. The merge and the independent channels are shown separately. Scale bars 50 μm.

Plasmatocytes of larvae infected by *L*. *boulardi* followed the same dynamics as those of uninfected larvae, but without the sudden increase late during infection. Activated plasmatocytes were present in the circulation already ten hours after infection. Their numbers stayed low until the 30 h time point. Then their count started to rise above the number of plasmatocytes and thereafter steadily increased. This coincided with the hatching of wasp larvae. After this time point, activated plasmatocytes were the predominant plasmatocyte population in circulation (Fig 2B), as well as in the sessile compartment (Fig 3). The final peak of activated plasmatocytes observed in infected larvae corresponded well to a similar increase in plasmatocytes in uninfected larvae (Fig 2A and 2B).

Lamelloblasts, prelamellocytes, and lamellocytes were rarely seen in uninfected larvae, but eight hours after infection lamelloblasts appeared for the first time, and during the following six hours their count rose to over 1000 cells per animal. Later, their numbers decreased and thereafter stayed more or less constant until the end of the time line experiment. Interestingly, the dynamics of prelamellocytes and lamellocytes followed that of lamelloblasts but with a delay of six and 16 h, respectively. Unlike lamelloblasts, the numbers of prelamellocytes and lamellocytes started to rise again 40 h after infection (Fig 2B’). In addition, we tested if temperature influences the induction of different blood cell types. The immune reaction of larvae grown at 25°C was similar to that of larvae grown at 29°C, however it was time-delayed due to the lower temperature (S6 Fig).

We used a reduced time line approach for *L*. *clavipes*- and *L*. *heterotoma*-infected *Me*/*w* larvae, focusing on the time points when a *L*. *boulardi* infection had the largest effects. In larvae infected by *L*. *clavipes*, the increase in total cell number was comparable to that after a *L*. *boulardi* infection (S5 Fig). Also the dynamics of the different cell types was similar, albeit delayed by four hours. The average numbers of prelamellocytes and lamellocytes never reached those after *L*. *boulardi* infection (S7B and S7B’ Fig). The total cell number of *L*. *heterotoma*-infected larvae was lower than after *L*. *boulardi* or *L*. *clavipes* infection (S5 Fig). Shortly after infection, it rose above the level of controls, but stayed relatively low thereafter. At the 48 h time point, the total cell count was lower than in controls. Lamelloblasts appeared in the circulation, but their numbers were low. Also the counts of activated plasmatocytes, prelamellocytes, and lamellocytes were reduced in comparison to larvae infected by the other wasp species. Activated plasmatocytes were not observed in the circulation until the 48 h time point (S7C and S7C’ Fig). Flow cytometry plots and representative microscopic images of hemocytes at selected time points after infection by the three wasp species and uninfected controls are presented in Fig 1C and S8 Fig. It is noteworthy that oviposition by all wasp species induced all blood cell populations with similar dynamics. This implies that the melanotic encapsulation response is stereotypical, and only shaped by the virulence factors of the infecting wasp species.

The outcome of a *L*. *boulardi* infection varied between individual *Drosophila* larvae. Thus, we recovered either living or killed wasp larvae from the hemocoel when we dissected fly larvae 36 to 50 h after infection. Similarly, melanized and non-melanized wasp eggs were present 24 h after a *L*. *clavipes* infection. This gave us the opportunity to test if the total cell number varied according to the outcome of the infection, thereby explaining the resistance to the parasite. However, in neither case did we see a significant difference in cell numbers (S5B and S5C Fig).

### Dynamics of sessile hemocytes over time

**Fig 4:**
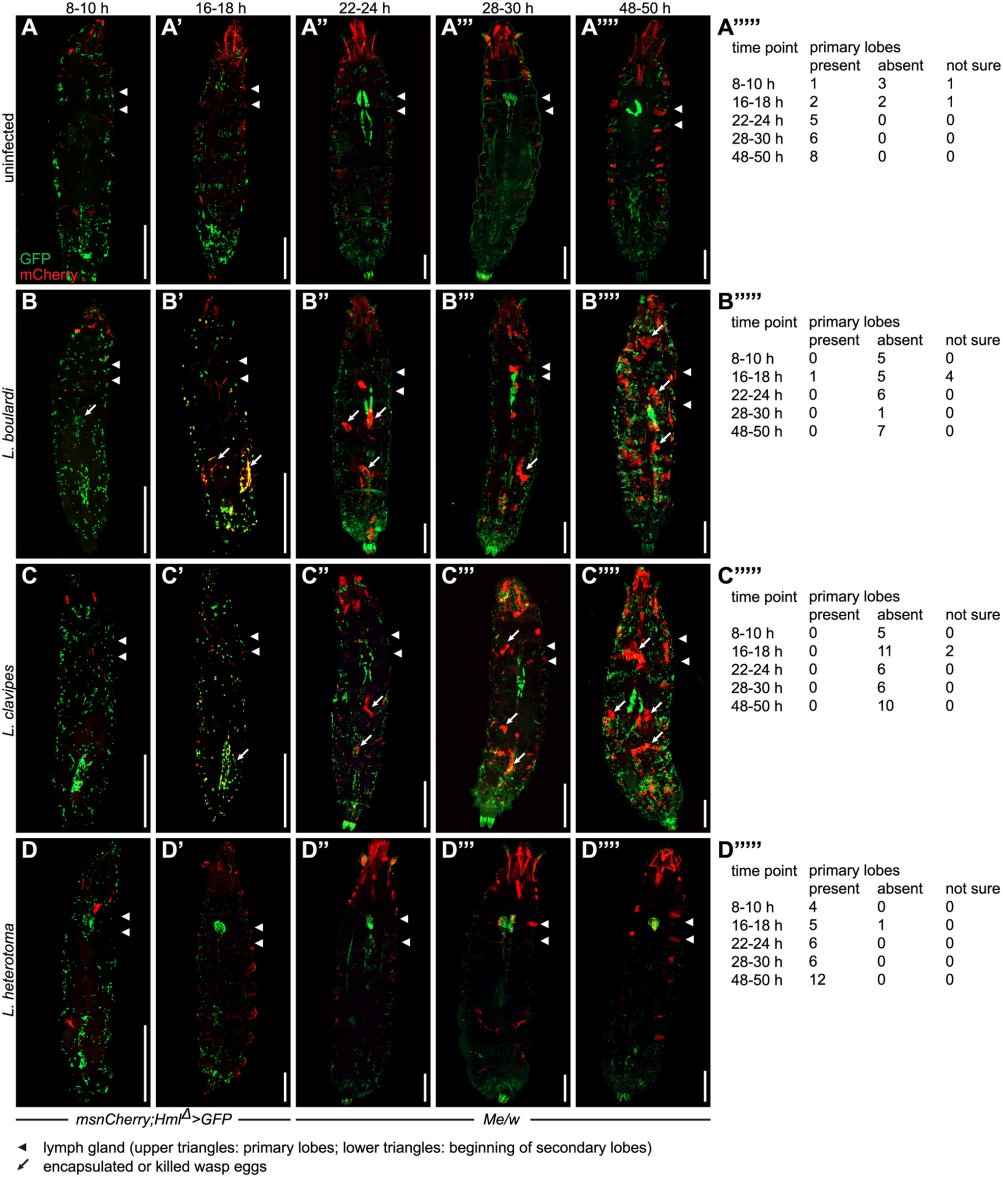
Chronological changes in the pattern of sessile cells in *Drosophila* larvae after a wasp infection. Sessile hemocyte populations including the lymph glands were imaged in *msnCherry*;*Hml* ^*Δ*^ *>GFP* and *Me/w* heterozygous control larvae (A-A”“), or at the indicated time points after infection by *L*. *boulardi* (B-B”“), *L*. *clavipes* (C-C”“), or *L*. *heterotoma* (D-D”“). The occurrence of primary lymph gland lobes at indicated time points in uninfected control larvae (A”“‘), *L*. *boulardi*-infected (B”“‘), *L*. *clavipes*-infected (C”“‘), and *L*. *heterotoma*-infected larvae (D”“‘). *msnCherry*;*Hml* ^*Δ*^ *>GFP* larvae were imaged 8–10 h and 16–18 h and *Me/w* larvae at the remaining time points. While GFP expression in uninfected and infected larvae was restricted to hemocytes, mCherry was also expressed in the pharyngeal muscles, the alary muscles, and in adjacent fibers of the lateral longitudinal muscles of the body wall. This ectopic mCherry expression increased with age. (A-A”“‘). After infection by *L*. *boulardi* and *L*. *clavipes*, mCherry expression in the lateral longitudinal muscles decreased, but became visible in pericardial cells. No change in ectopic mCherry expression was observed after *L*. *heterotoma* infection (B-D”“). In uninfected larvae, sessile plasmatocytes were clearly visible by their GFP expression. The primary and secondary lymph gland lobes were always visible at the later time points, albeit sometimes obscured due to the imaging method (A-A”“). 8–10 h after an infection by *L*. *boulardi* and *L*. *clavipes*, no obvious change in the pattern of sessile cells had occurred (B, C). The primary lymph gland lobes were never visible (B”“‘, C”“‘). At 16–18 h, fluorescent blood cells outlined the shape of the wasp eggs and started to express mCherry locally on or in the vicinity of the wasp eggs. No systematic loss of sessile blood cells was observed (B’, C’). The primary lymph gland lobes were predominantly absent (B”“‘, C”“‘). At 22–24 h after an infection by *L*. *boulardi* and *L*. *clavipes*, the mCherry expression became more visible and localized to the wasp eggs, but mCherry-positive cells formed nodules that were not always connected to wasp eggs (B”, C”). Primary lymph gland lobes were always absent while the secondary lobes were visible (B”, C”). At the two final time points after a *L*. *boulardi* and *L*. *clavipes* infection, total cell numbers had increased. Encapsulated wasp eggs or larvae were clearly visible. In addition, nodules had formed in areas where no wasp eggs or larvae were present. Secondary lymph gland lobes were hypertrophied (B”‘-B”“, C”‘-C”“), whereas primary lymph gland lobes were always absent (B”“‘, C”“‘). A *L*. *heterotoma* infection led to a decrease in sessile blood cell numbers. No mCherry-positive nodules were formed during the course of the infection. The primary lymph gland lobes were present and contained mCherry-positive cells starting from 22–24 h. Secondary lymph gland lobes were not visible at the 28–30 h nor at the 48–50 h time points (D-D”“‘). Scale bars 500 μm.

It was reported previously that hemocytes leave the sessile islets and start circulating after a wasp infection [17,18]. We therefore imaged control larvae and larvae infected by the three wasp species at different time points after infection to have a closer look at the pattern of sessile cells and the lymph glands (Fig 4). The fluorescent signals of the *eaterGFP* and *msnCherry* reporters were weak until approximately 22 h after infection, presumably due to the combination of a long maturation time of the fluorophores and a comparatively low magnification. The signal was stronger when we expressed *UAS*-*GFP* with the hemocyte-specific *Hml* ^*Δ*^-*GAL4* driver (*Hml* ^*Δ*^>*GFP*), which is expressed from an early time point in development [12,42]. For this reason, we imaged the offspring of crosses of *msnCherry* to *Hml* ^*Δ*^>*GFP* (*msnCherry*;*Hml* ^*Δ*^>*GFP*) at 8 and 16 h after infection. At all other time points, we used heterozygous *Me*/*w* reporter larvae.

In uninfected larvae, we were able to detect *Hml* ^*Δ*^>*GFP* in the lymph glands of only one out of five cases at the 8–10 h time point and in two out of five cases at the 16–18 h time point (Fig 4A–4A”“). Different wasp species had varying effects on the lymph glands. Early after parasitization by *L*. *boulardi* and *L*. *clavipes*, the primary lymph gland lobes disappeared while the secondary lobes were enlarged later. This was best visible before the application of structured illumination as described in Materials and Methods (see numbers in Fig 4A”“‘, 4B”“‘ and 4C”“‘). In contrast, the primary lobes were always visible after a *L*. *heterotoma* infection, and they contained lamellocytes starting from the 22 h time point, whereas the secondary lobes disappeared (Fig 4D”“‘). This was in contrast to a previously published report where a *L*. *heterotoma* infection induced apoptosis in the lymph glands [43].

We did not observe any major effect on the sessile hemocyte population 8 or 16 h after infection by any of the three wasp species. 48–50 h after *L*. *boulardi* and *L*. *clavipes* infections, the number of sessile plasmatocytes seemed to increase and mCherry-positive nodules of lamellocytes became visible (Fig 4B”–4B”“ and 4C”–4C”“). Under these conditions, the previously observed mobilization of sessile cells [17,18] was not apparent. A *L*. *heterotoma* infection induced a progressive loss of sessile plasmatocytes, and lamellocytes were rarely seen among sessile cells (Fig 4D”–4D”“).

As noted previously [44] the *msnCherry* reporter was also ectopically expressed in the pharyngeal musculature, in one to three adjacent lateral transverse muscles on each side of most segments, and in the alary muscles. After infection by *L*. *boulardi* and *L*. *clavipes*, mCherry expression became activated in pericardial cells, while its expression in the lateral transverse musculature abated (Fig 4B–4B”“ and 4C–4C”“). A similar effect was previously observed in the *Toll* ^*10b*^ gain-of-function mutant [44]. No change was observed after an infection by *L*. *heterotoma* (Fig 4D–4D”“).

### Hemocytes on wasp eggs

**Fig 5:**
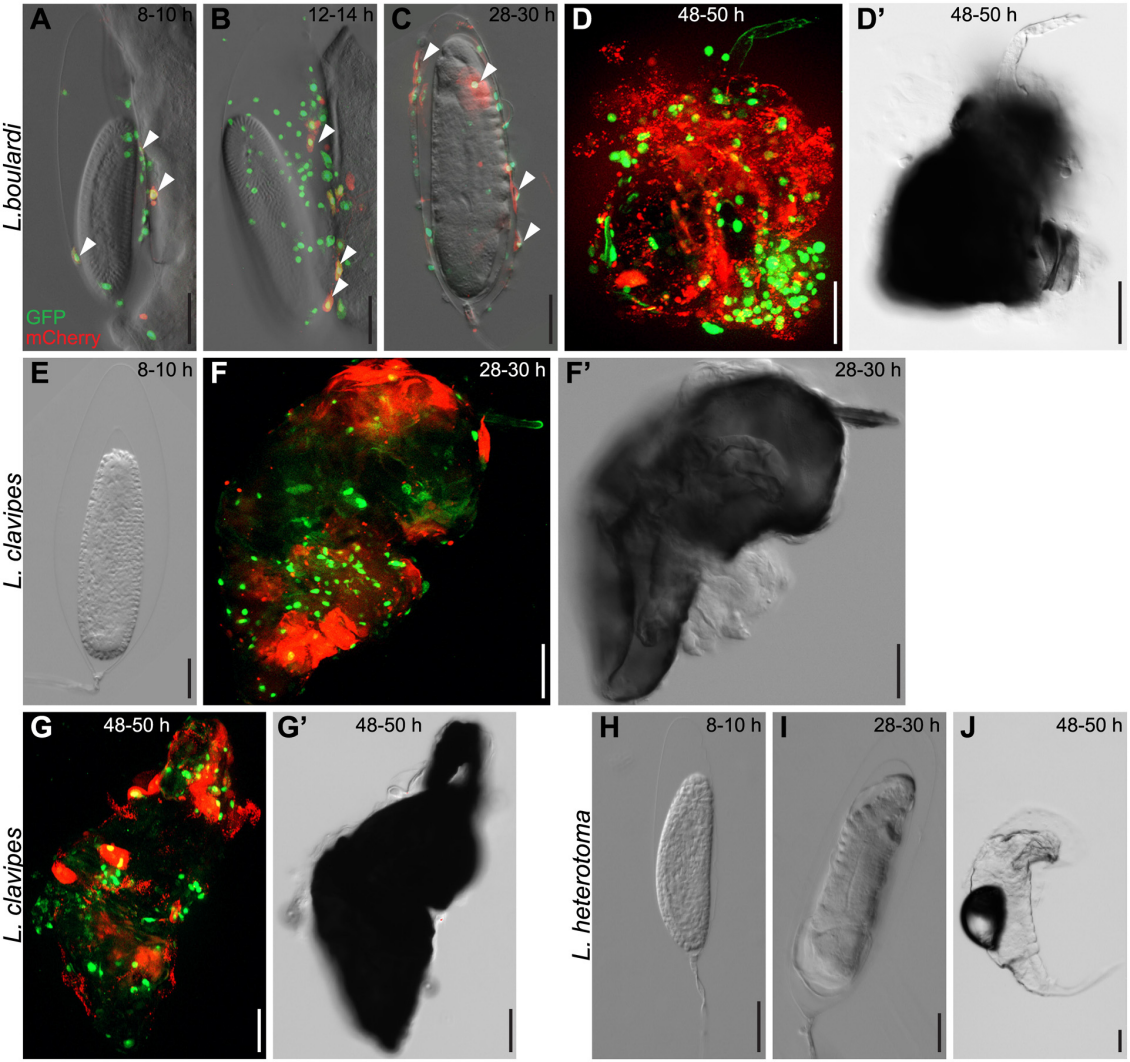
Chronological events during the cellular immune response on the surface of wasp eggs. *Me/w* heterozygous *Drosophila* larvae were infected by (A-D’) *L*. *boulardi*, (E-G’) *L*. *clavipes* or (H-J) *L*. *heterotoma*, and wasp eggs and wasp larvae were dissected and imaged at the indicated time points. *L*. *boulardi* eggs were always attached to the gut. Already 8–10 h after infection, plasmatocytes were visible on the wasp eggs and some started to express mCherry, transforming into activated plasmatocytes. The number of plasmatocytes and activated plasmatocytes on the wasp egg increased during the course of infection. In most cases, *L*. *boulardi* wasp larvae hatched around 32 h after infection, and some of them were later killed by the cellular immune system. No hemocytes attached to *L*. *clavipes* eggs early after infection. Eggs were encapsulated and melanized 28–30 h after infection, and wasp larvae rarely hatched from these eggs. No hemocytes ever attach to the freely floating eggs of *L*. *heterotoma*, and the wasp larvae of this species hatched around 38 h after infection.

Next we imaged wasp eggs of the different wasp species at various time points after infection. The events on the wasp egg differed depending on the wasp species. After a *L*. *boulardi* infection, plasmatocytes and activated plasmatocytes were found attached to wasp eggs eight to ten hours after infection (Fig 5A). Their numbers increased 12–14 h and type II lamellocytes formed and started to spread (Fig 5B). Type II lamellocytes were fully spread 28–30 h after infection. No melanotic encapsulation reaction had occurred yet, and type I lamellocytes were rarely present on wasp eggs at this point (Fig 5C). Approximately 32 h after infection, *L*. *boulardi* larvae hatched and the cellular immune system mounted a melanotic encapsulation response against the wasp larvae. Fig 5D and 5D’ show an encapsulated wasp larva 48–50 h after infection, with lamellocytes and plasmatocytes participating in the formation of the capsule.

In *L*. *clavipes*-infected larvae, hemocytes did not attach to the wasp egg early during infection (Fig 5E). Instead, they formed loose networks around wasp eggs. These networks were too delicate to dissect and therefore difficult to image. As soon as lamellocytes were present, 22–24 h after infection, melanotic encapsulation ensued rapidly. Both lamellocytes and plasmatocytes were involved in forming the capsule. Wasp eggs were fully encapsulated 28–30 h after infection (Fig 5F and 5F’) and the capsule became darker with time (Fig 5G and 5G’). The immune system of fly larvae was not able to attack wasp eggs or larvae after a *L*. *heterotoma* infection at any time point (Fig 5H–5J).

### Wasp infection induces a demand-adapted hematopoiesis

**Fig 6:**
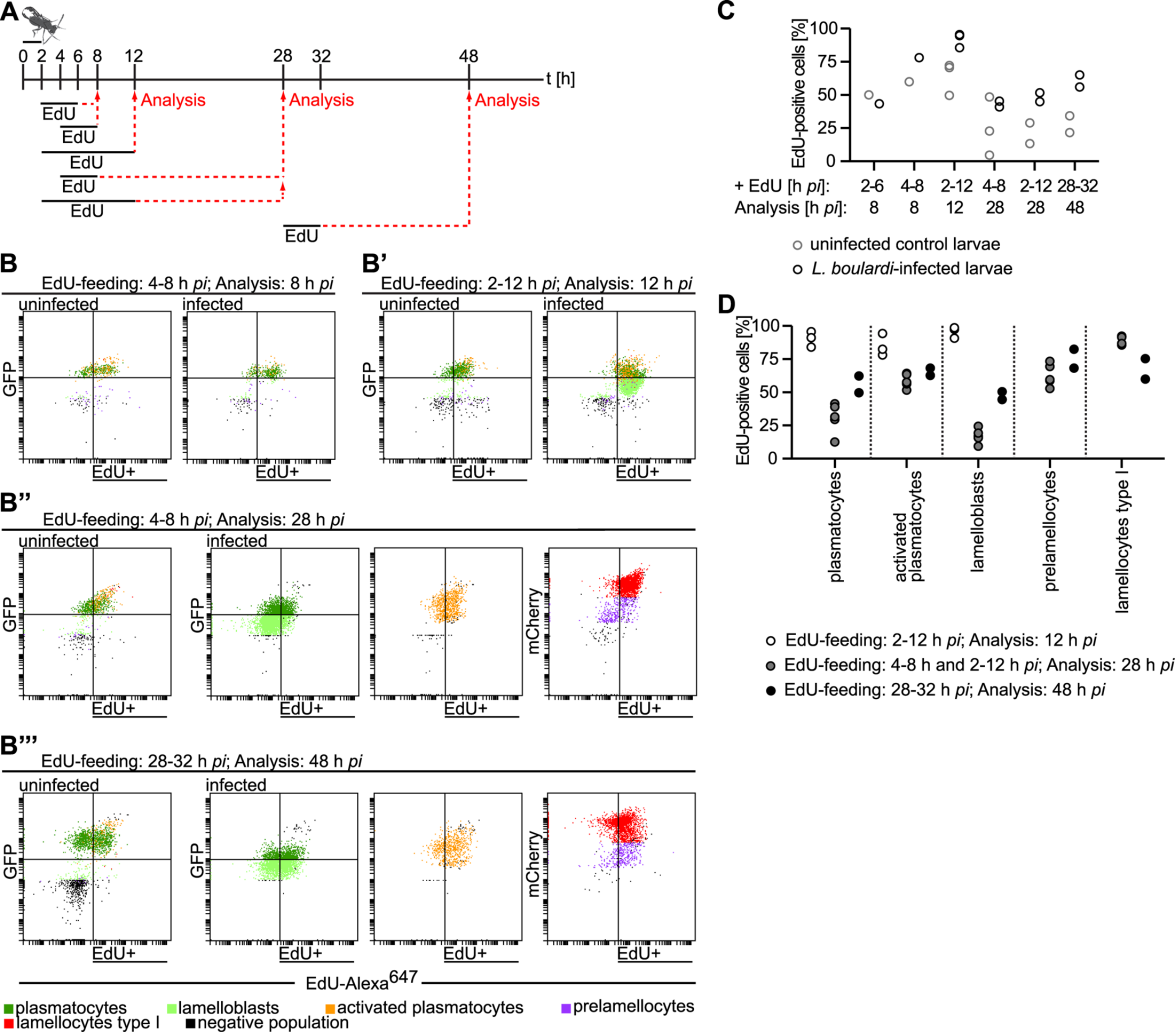
Proliferation is necessary for the demand-adapted hematopoiesis in *Drosophila* larvae after a wasp infection. (A) The experimental design of EdU feeding and subsequent analysis of hemocytes by flow cytometry of age-matched uninfected and infected larvae. Larvae were infected by *L*. *boulardi* for two hours, moved to EdU-containing food at the indicated time points and durations, and finally the flow cytometry analysis was carried out accordingly. (B-B”‘) Representative scatter plots of hemocytes of control and wasp-infected larvae after EdU-feeding. EdU was visualized by Alexa^647^ labeling. (B) EdU feeding 4–8 h and analysis 8 h after infection. In wild-type control larvae approximately half of the hemocytes incorporated EdU, whereas the EdU incorporation rate slightly increased in infected larvae. (B’) EdU-feeding 2–12 h and analysis 12 h after infection. Almost every lamelloblast, plasmatocyte, and activated plasmatocyte incorporated EdU. (B”) EdU-feeding: 4–8 h and analysis 28 h after infection. Plasmatocytes, activated plasmatocytes, lamelloblasts, prelamellocytes and lamellocytes are shown separately. The amount of EdU incorporating plasmatocyte types after infection approximately equaled that of controls, whereas many of the infection-induced prelamellocytes and lamellocytes incorporated EdU. (B”‘) EdU feeding: 28–32 h and analysis 48 h after infection. Plasmatocytes, activated plasmatocytes, lamelloblasts, prelamellocytes and lamellocytes are shown separately. Slightly more plasmatocytes and lamelloblasts of infected larvae were EdU-positive, while the number of EdU-positive activated plasmatocytes, prelamellocytes and lamellocytes was again high. (C) Percentages of EdU-positive total hemocyte numbers of wasp-infected and control larvae. (D) Quantification of EdU incorporation experiments in (B-B”‘) according to hemocyte types. One to three replicates were conducted for each EdU- feeding scheme. pc—plasmatocytes.

The sudden appearance of lamelloblasts was the most striking observation in the time line experiment. Their numbers increased from zero to over 1000 cells per larva within only six hours (Fig 2B’). This increase was most likely due to the proliferation of lamelloblasts or their precursors. We therefore devised experiments to investigate cell division after infection. We placed *Me*/*w* larvae together with *L*. *boulardi* females for two hours for infection and then applied different 5-ethynyl-2′-deoxyuridine (EdU) feeding schemes to test which cell types were capable of division early and late after a wasp infection (Fig 6A). The gating strategy of EdU-Alexa^647^ combined with GFP and mCherry expression can be found in S9 Fig. Eight hours after infection, total cell numbers were slightly elevated in infected larvae (S5 Fig), lamelloblasts were seen in the circulation for the first time (Fig 2B’), and type II lamellocytes appeared on the wasp eggs (Fig 5A). Therefore we fed larvae with EdU at different early time intervals and then analyzed EdU incorporation eight hours after infection to find out the earliest time point of infection-induced cell division (Fig 6A). After the first EdU feeding scheme, two to six hours on EdU food, approximately half of the hemocytes of control and infected larvae had incorporated EdU (Fig 6C). After the second feeding scheme, four to eight hours on EdU food, a larger number of hemocytes of infected larvae were EdU positive than of controls (Fig 6B and 6C). We observed the highest number of lamelloblasts 14 h after infection (Fig 2B’). Therefore, we predicted that just prior to that time point, lamelloblasts would be dividing at their highest rate. Thus, we fed larvae on EdU two to 12 h and dissected hemocytes 12 h after infection (Fig 6A). At this time point, lamelloblasts and all plasmatocyte subtypes had incorporated EdU at a high rate (Fig 6B’, 6C, and 6D). Thus, a demand-adapted increase in hemocyte proliferation was already well under way at eight hours after infection, and most plasmatocytes and lamelloblasts had undergone division at least once by 12 h (Fig 6C).

**Fig 7:**
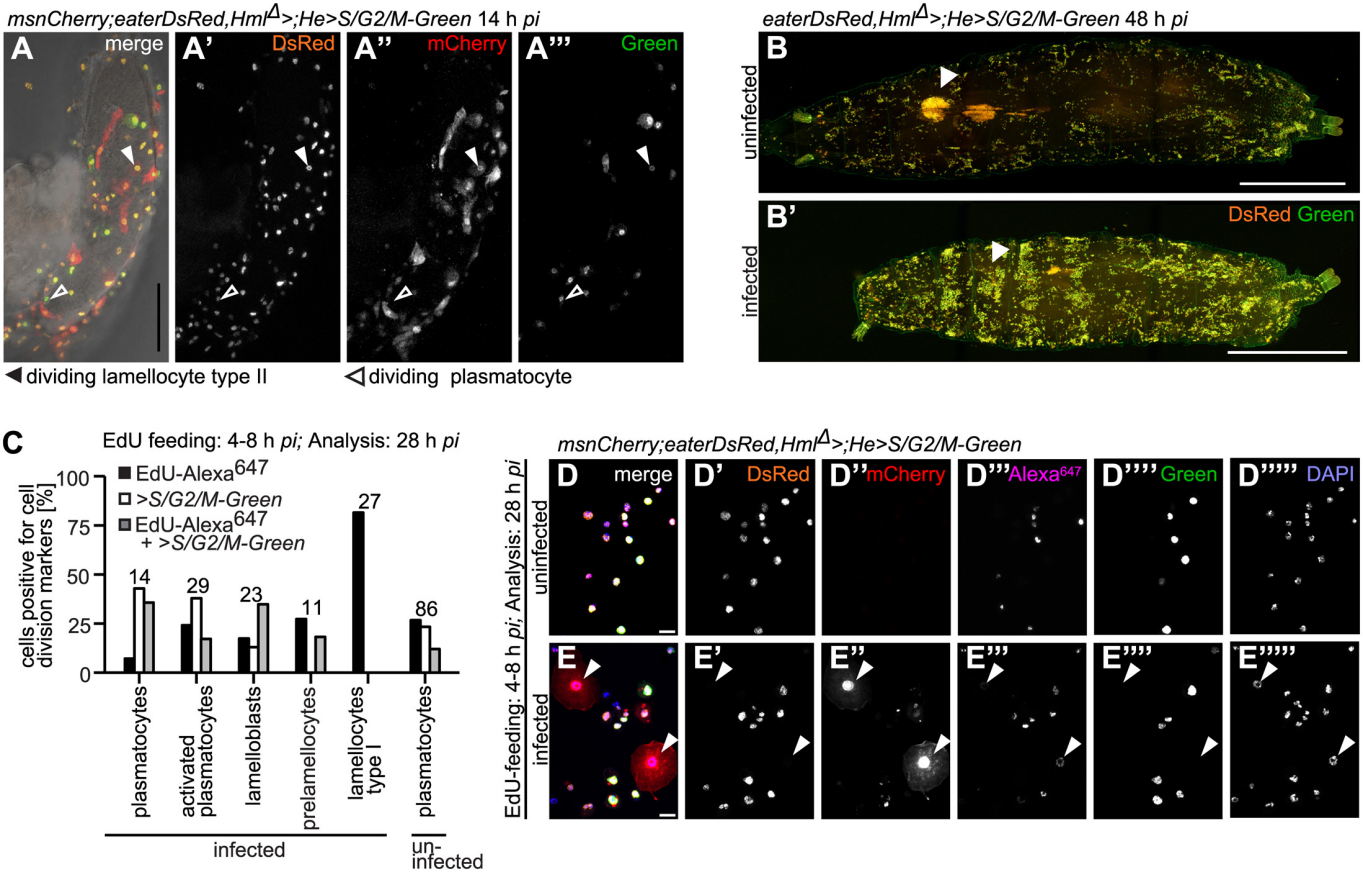
All larval hemocyte types are able to divide, except for lamellocytes. (A-A”‘) Hemocytes were able to divide on wasp eggs. Arrowheads point to an example of a dividing activated plasmatocyte and a dividing plasmatocyte. Scale bars 50 μm. (B-B’) Sessile blood cells divided in control and *L*. *boulardi*-infected larvae 48 h after infection. *eaterDsRed;Hml* ^*Δ*^ *>* was crossed with *w;He>S/G2/M-Green* to obtain *eaterDsRed;Hml* ^*Δ*^ *>;He>S/G2/M-Green*. Observe that due to the genotype only plasmatocytes are visible after infection! The number of sessile cells increased after a wasp infection. In the wasp-infected larva, the primary lymph gland lobes were no longer present, whereas they were still visible in the control larva. Arrowheads point to the locations of the primary lymph gland lobes. Scale bars 100 μm. (C) Quantification of dividing cell types after EdU feeding 4–8 h and analysis 28 h after infection of uninfected and *L*. *boulardi*-infected larvae. Hemocytes of offspring of crosses of *msnCherry;eaterDsRed*,*Hml* ^*Δ*^ *>* to *w;He>S/G2/M-Green* were imaged and analysed for the incorporation of EdU-Alexa^647^, and/or the expression of *S/G2/M-Green*. The total numbers of evaluated cells per cell type are indicated above the bar plots of each hemocyte type (pc: plasmatocytes). All cell types incorporated EdU. All cell types but lamellocytes expressed Green. All cell types except for lamellocytes incorporated EdU and simultaneously expressed *>S/G2/M-Green*. The gating strategy is explained in S9 Fig. (D-E”“‘) Representative images of cells analyzed in (C). Arrowheads point to lamellocytes. None of the lamellocytes expressed Green (E”“), but one lamellocyte that had incorporated EdU is shown (E”‘). Scale bars 50 μm.

As type II lamellocytes were scarce in circulation, we investigated if these cells were able to divide on wasp eggs. We used *UAS*-*S*/*G2*/*M*-*Green* under the control of the combined hemocyte-specific drivers *Hml* ^*Δ*^-*GAL4* and *He*-*GAL4* to mark dividing cells. *S*/*G2*/*M*-*Green* is an *in vivo* cell cycle reporter that labels cells in the S-, G2-, and M-phases [45,46]. Fig 7A–7A”‘ show that plasmatocytes and type II lamellocytes were dividing while attached to a wasp egg. At 48–50 h after infection, sessile plasmatocyte numbers seemed to have increased after a *L*. *boulardi* and a *L*. *clavipes* infection in comparison to controls (Fig 4A”“, 4B”“ and 4C”“). We used *S*/*G2*/*M*-*Green* in combination with *eaterDsRed* to assess whether sessile plasmatocytes were dividing. Within the sessile population of controls and infected larvae a large proportion of plasmatocytes divided (Fig 7B and 7B’).

### Plasmatocytes mature into activated plasmatocytes after infection and transdifferentiate into type II lamellocytes on the wasp egg

Because genetic tools have not yet been developed for the newly discovered cell types, we deduced the relationships between these classes by a different approach. We reasoned that changes in marker expression must be gradual. By sampling the population at close enough time points, we expected all hemocyte classes to be linked to their precursors via intermediate forms. In this way we could trace hematopoiesis to two parallel lineages, the plasmatocyte and lamellocyte lineages, each originating from a different class of plasmatocyte-like cells. In addition to changes in the expression levels of fluorescent markers, both forward and side scatter increased the further advanced the cells were in each lineage, suggesting that they became larger and more granular (S4E–S4E” Fig). In the plasmatocyte lineage plasmatocytes gave rise to two infection-induced cell types; activated plasmatocytes and type II lamellocytes. The continuity of the cell population from the plasmatocyte gate into the activated plasmatocyte gate (Fig 1C) suggested that plasmatocytes developed into activated plasmatocytes by retaining GFP expression and gradually accumulating cytoplasmic mCherry-positive foci. 30–32 h after infection, activated plasmatocyte counts increased in synchrony with the hatching of wasp larvae, whereas plasmatocyte numbers remained constant (Fig 2B). This implied that both cell types are able to proliferate. Feeding larvae on EdU-containing food 28–32 h after infection and dissecting them 16 h later (Fig 6A) revealed that plasmatocytes and activated plasmatocytes had incorporated EdU at the same rate (Fig 6B”‘ and 6D). In addition, we compared the granularity and the size of plasmatocytes and activated plasmatocytes 48 h after infection. The plasmatocytes of infected larvae were smaller and less granular than activated plasmatocytes and the plasmatocytes of non-immune challenged larvae (S4D–S4G’ Fig). Therefore, we concluded that plasmatocytes matured into activated plasmatocytes, both cell types proliferated, and these cell types differed in size and granularity.

**Fig 8:**
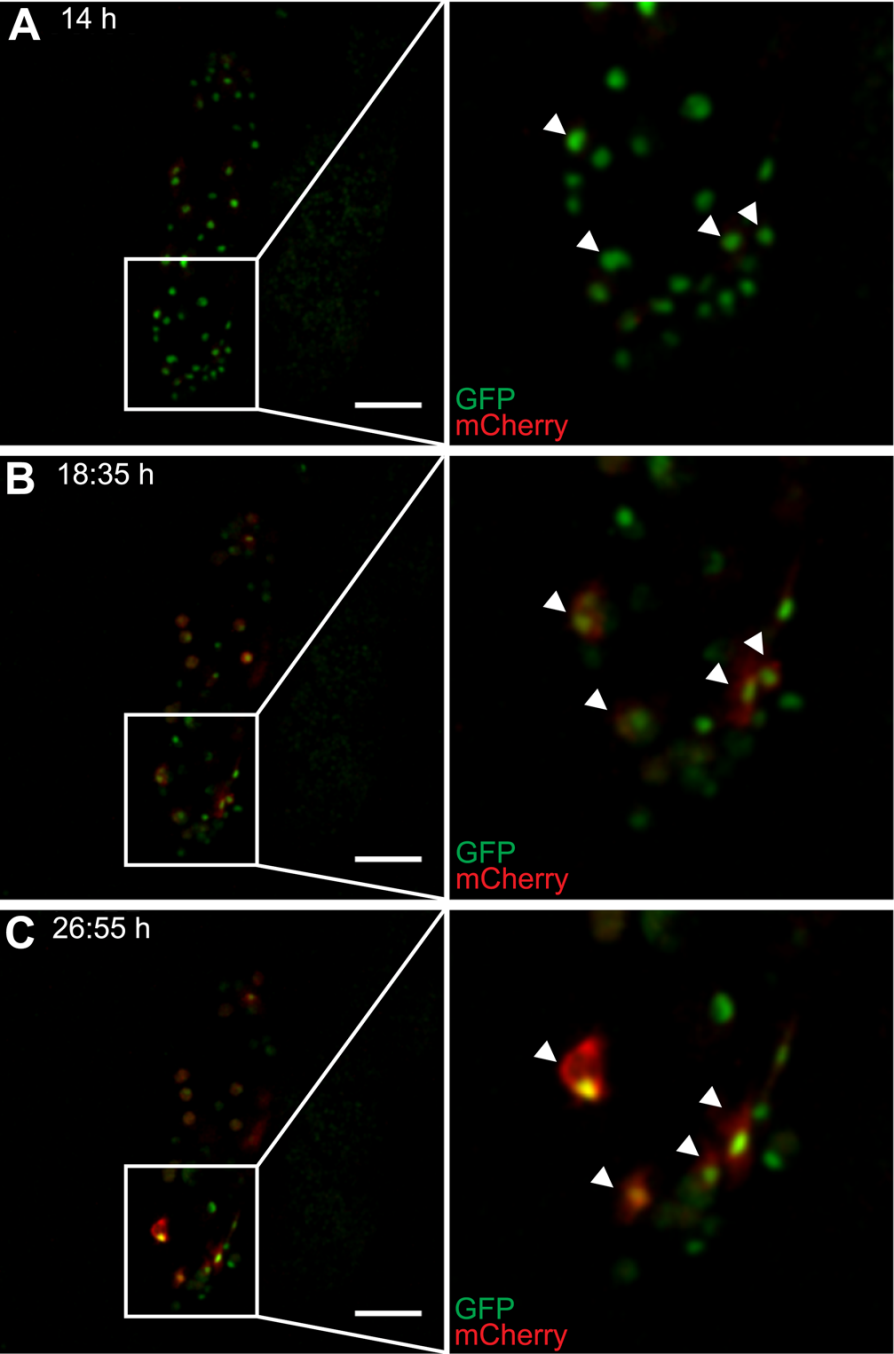
Activation of plasmatocytes on wasp eggs. (A-C) Still images of hemocytes on the surface of a wasp egg, taken from a time lapse experiment of *Me/w* larvae infected by *L*. *boulardi*. Wasp eggs with attached hemocytes were dissected out 14 h after infection and the attached hemocytes are shown at representative time points after infection: (A) 14 h, (B) 18:35 h and (C) 26:55 h. Plasmatocytes started to express and to continuously increase their expression levels of mCherry, spread on the wasp egg, and thereby transformed into type II lamellocytes. The corresponding time-lapse video can be found at S1 Video. Scale bars 50 μm.

In addition, soon after plasmatocytes adhered to the parasite, they started to express mCherry, increased in size, and turned into type II lamellocytes by transdifferentiation (Fig 8A–8C, S1 Video). At this stage, their shape became reminiscent of lamellocytes, but they never lost their plasmatocyte identity, as defined by the expression of the GFP marker (Figs 5C and 8A–8C).

### Lamelloblasts are the founder population of circulating lamellocytes

We noted that lamelloblasts appeared eight hours after oviposition, followed by prelamellocytes at 14 h and finally lamellocytes at 22 h (Fig 2B’). These three hemocyte populations formed one contiguous streak in the two-dimensional scatter plots (Fig 1C). This suggested that lamelloblasts were precursors of lamellocytes. Therefore, early feeding of EdU would generate EdU-positive precursors that in turn would give rise first to EdU-positive prelamellocytes and then to lamellocytes. To test this prediction, we fed larvae with EdU at two early time points after infection (4–8 h and 2–12 h), put them back on normal food, and analyzed hemocytes 28 h after infection (Fig 6A). At 28 h, approximately half of all hemocytes of infected larvae had incorporated EdU (Fig 6B” and 6C). Because the effects of feeding with EdU in these two experiments were similar, we pooled the results when looking at individual cell types. Indeed, 50–75% of prelamellocytes and 80–100% of lamellocytes were EdU-positive. The fraction of EdU-positive plasmatocytes, activated plasmatocytes, and lamelloblasts was reduced in comparison to the previous time point (Fig 6B” and 6D).

Lamellocytes have so far been regarded as terminally differentiated and non-dividing cells [47], but we found that most lamellocytes were EdU-positive (Figs 6D and 7A). To ascertain that the incorporation of EdU was due to an earlier cell division event in precursor cells, namely lamelloblasts and prelamellocytes, we double-labeled hemocytes with EdU and *Hml* ^*Δ*^ *;He>S/G2/M-Green*, using the same feeding scheme as described above. No lamellocyte ever expressed Green alone or in combination with EdU while almost all lamellocytes had incorporated EdU. This indicated that lamellocytes themselves were not dividing nor replicating DNA, but did indeed develop from dividing precursor cells. As shown before, lamelloblasts and all plasmatocyte subtypes, but also prelamellocytes were proliferating (Fig 7A–7C). We also found EdU-positive prelamellocytes during a later feeding scheme (Fig 6A, 6B”‘ and 6D). This suggests that prelamellocytes were still dividing 28–32 h after infection, giving rise to non-dividing lamellocytes. We corroborated our FACS results by imaging EdU-labeled hemocytes of three feeding schemes and obtained similar results (S10 and S11 Figs).

### Loss of *edin* in the fat body reduces the number of lamelloblasts

**Fig 9:**
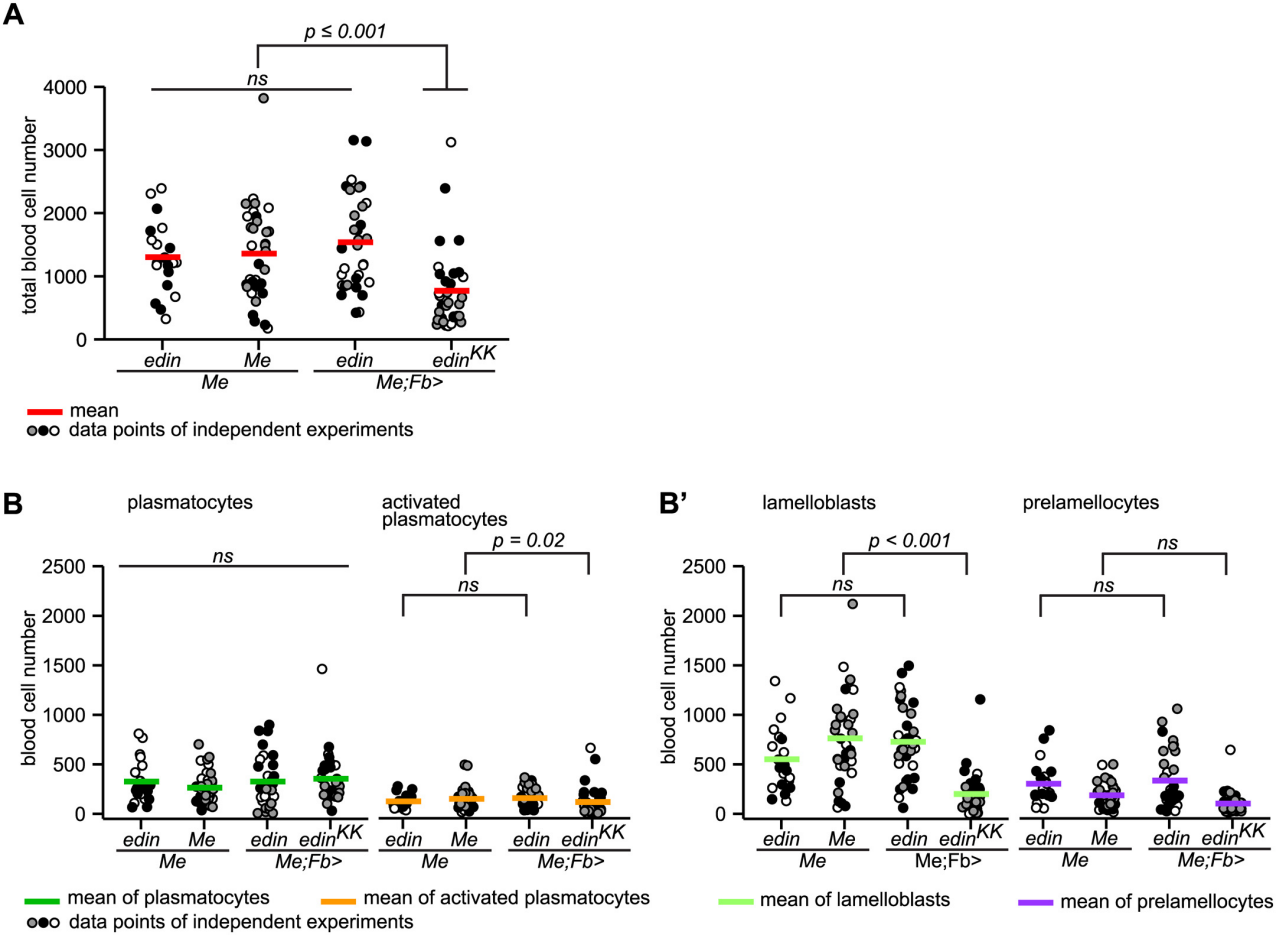
Suppression of *edin* expression in the fat body inhibits the formation of lamelloblasts. (A) Knock-down of *edin* in the fat body reduced total hemocyte numbers 14 h after infection by *L*. *boulardi* (ANOVA: replicate*genotype: F = 1.983, p = 0.121, df = 3; replicate: F = 0.746, p = 0.39, df = 1; genotype: F = 10.923, p < 0.001, df = 3; for pairwise comparisons we used Tukey`s HSD). Three independent experiments are shown for the genotypes *Me*, *Me;Fb>edin*, and *Me;Fb>edin* ^*KK*^, and two for the genotype *Me;edin*. Hemocyte numbers of each experiment are represented by dots in different shades and the mean hemocyte numbers by red bars. At least ten larvae were bled per genotype and experiment. (B) Knock-down of *edin* in the fat body reduced the number of lamelloblasts (Kruskal-Wallis rank sum test: genotype: H = 44.913, p < 0.001, df = 3; for pairwise comparisons we used the independent two-group Mann-Whitney U Test) 14 h after infection by *L*. *boulardi*. Identical experiments as in (A), but the cell numbers for each genotype are plotted separately. The mean cell numbers for each cell type are plotted as colored bars. *ns*—not significant.

We recently showed that the peptide Edin is required for the normal wasp-induced recruitment of plasmatocytes into the circulation from the sessile compartment. When we suppressed *edin* expression in the fat body, total cell numbers were reduced 14 h after infection, and at 48 h total plasmatocyte numbers did not rise above control levels [48]. We now explored in detail how the different hemocyte classes were affected when we manipulated *edin* expression. We suppressed or overexpressed *edin* in the fat body and assessed blood cell numbers by flow cytometry 14 h after infection by *L*. *boulardi*. As expected, total blood cell numbers were significantly reduced when an *edin* RNAi construct was expressed in the fat body, while the effect of *edin* overexpression, if any, was not statistically significant (Fig 9A). The effect of *edin* RNAi was most evident with lamelloblasts. Their number was reduced to less than half (Fig 9B’). Also prelamellocytes were reduced in *edin*-depleted larvae (Fig 9B’), but few prelamellocytes had formed at this time point and the effect of *edin* suppression was not statistically significant. Activated plasmatocytes were slightly reduced when *edin* was suppressed (Fig 9B).

### Comparison of hemocyte subclasses defined by fluorescent markers and monoclonal antibodies

**Fig 10:**
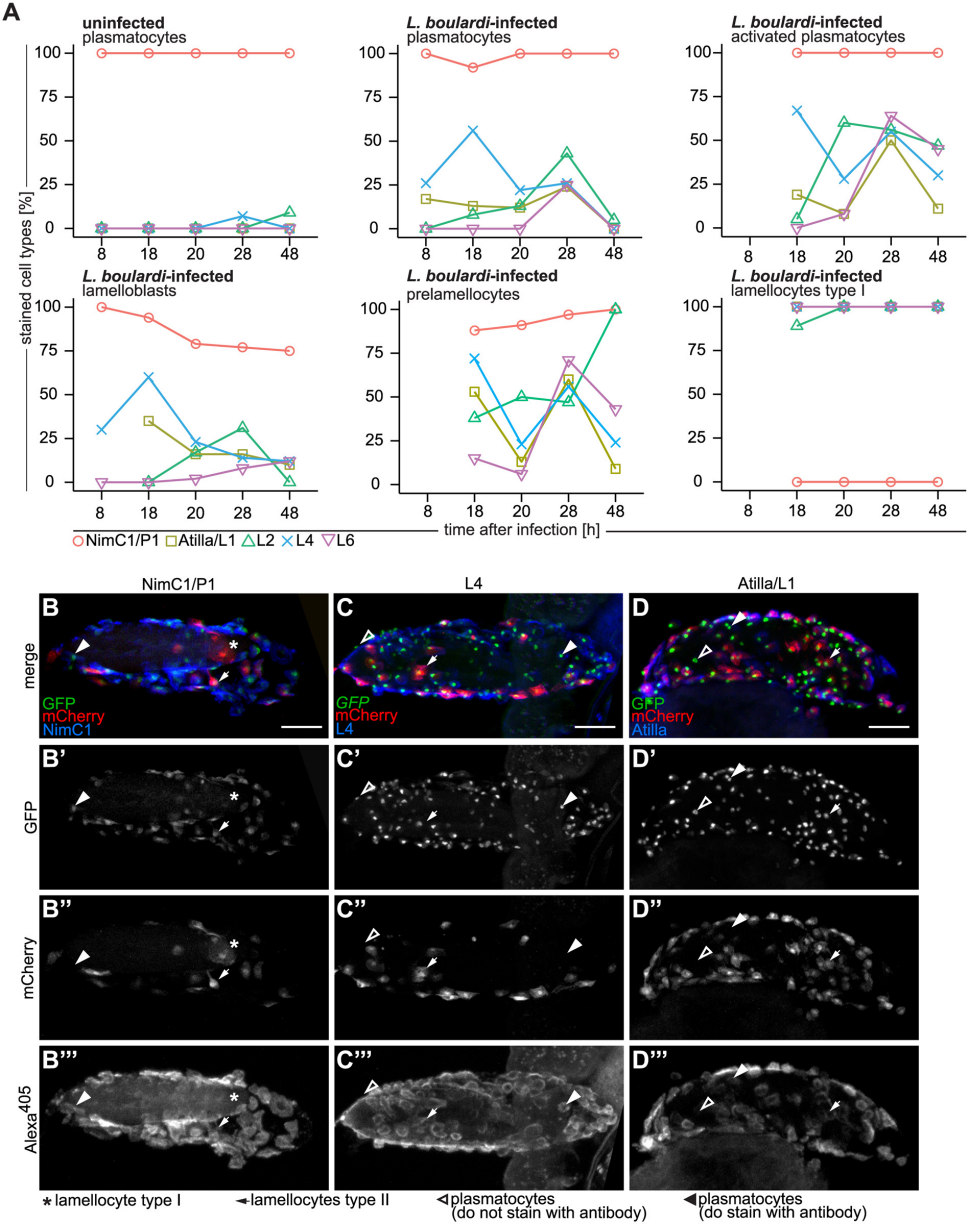
Antibody staining of hemocytes fixed on glass slides and on *L*. *boulardi* eggs. (A) Staining of *Me*-expressing hemocytes of age-matched control and *L*. *boulardi*-infected larvae with NimC1/P1, Atilla/L1, L2, Myospheroid/L4, and L6 antibodies at 8, 18, 20, 28, and 48h after infection. Individual hemocyte types are shown in separate graphs. Hemocytes of uninfected control larvae were primarily plasmatocytes and stained mainly with NimC1/P1 antibody at all time points. All blood cells of infected larvae stained with NimC1/P1 as long as they expressed *eaterGFP* (> 75% in all cell types). The percentage of expression of NimC1 was lowest in lamelloblasts from 20 h after infection onwards. Lamellocytes never expressed NimC1 and expressed all of the lamellocyte antigens. After infection, all blood cell types stained to some extent with lamellocyte markers. The L4 antigen was the first and the L6 antigen the last to be expressed by circulating hemocytes. Some cell types were rare at certain time points. A missing data point indicates that fewer than ten cells were counted, and these data were excluded from the analysis. (B-D”‘) Hemocytes of Me/w on *L*. *boulardi* eggs stained with (B-B”‘) NimC1/P1, (C-C”‘) L4, and (D-D”‘) Atilla/L1 14 h after infection. Alexa^405^ was used as the secondary antibody label. All channels are shown separately and as a merge. Scale bars 50 μm. The NimC1/P1 antibody did not recognize lamellocytes on the wasp egg, but did recognize plasmatocytes and activated plasmatocytes. Myospheroid/L4 and Attila/L1 stained most plasmatocytes and activated plasmatocytes on the wasp egg.

Monoclonal antibodies have been widely used to define different *Drosophila* hemocyte populations, as well as to identify proteins with hemocyte-specific functions [28]. The P1 antibody recognizes a phagocytosis receptor, NimC1, which is known to be expressed by cells with plasmatocyte morphology. Several antibodies, Atilla/L1, L2, Myospheroid/L4, and L6 (the L-antibodies), detect different proteins on lamellocytes [4,49,50]. We were therefore interested to see how these antigens were distributed among the hemocyte classes described here. We used these antibodies to stain hemocytes from uninfected and *L*. *boulardi*-infected *Me*/*w* larvae at different time points. As expected, NimC1 was expressed by all *eaterGFP*-positive cells derived from uninfected larvae. A small proportion of these cells also expressed the L2 and L4 antigens at the 28 and 48 h time points (Fig 10A).

In infected animals, lamellocytes were always NimC1-negative and were stained with all L-antibodies. All other hemocyte types consistently expressed NimC1 (Fig 10A and S12 Fig), but the tempo-spatial patterns of L-antigen expression in them were more complex. Generally only a minority of plasmatocytes and lamelloblasts expressed these antigens (< 30%). L6 was previously exclusively detected in mature lamellocytes [21]. In our experiments, L6 was also the least prevalent L-antigen to be expressed in hemocyte types other than lamellocytes, but we did observe some L6-positive activated plasmatocytes and prelamellocytes (Fig 10A, S12L–S12O Fig, Table 1). L4 was expressed in more than 50% of plasmatocytes, activated plasmatocytes, and lamelloblasts already 18 h after infection. At 28 h after infection, half of the activated plasmatocytes were positive for all L-antigens, but the number of stained cells decreased at the 48 h time point (Fig 10A). A high but variable fraction of prelamellocytes stained with L-antibodies. Except for L2, the expression of these markers all dropped at 48 h. A similar trend was obvious in the different plasmatocyte lineage cells.

**Table 1:** Antibody staining of prelamellocytes and lamellocytes type II at 18 h after infection by *L*. *boulardi* wasps.

Cell type	Antibody	Stained [%]	Total cells
Prelamellocyte	NimC1/P1	82	17
	Atilla/L1	100	7
	L2	50	16
	L4	100	8
	L6	30	10
Lamellocyte type II	NimC1/P1	100	10
	Atilla/L1	83	6
	L2	0	9
	L4	100	8
	L6	0	4

Prelamellocytes and type II lamellocytes were scarce in the circulation, and were detected in sufficient numbers only 18 h after infection. All type II lamellocytes were P1- and L4-positive, while being negative for L2 and L6, and 83% expressed L1 (Table 1, S12K–S12O’ Fig). Also on the wasp egg type II lamellocytes expressed P1 (Fig 10B–10B”‘), L1 (Fig 10D–10D”‘), and L4 (Fig 10C–10C”‘). Plasmatocytes on the wasp eggs showed varying expression of L1 and L4 and expressed P1 (Fig 10B–10D”‘). Prelamellocytes were L1 and L4 positive, and a large majority of them expressed also NimC1 (83%). L2 and L6 were expressed in 50% and 30% of prelamellocytes, respectively (Table 1).

In conclusion, L1, L2, L4, and L6 were detected in all lamellocytes, and to a variable extent in their likely precursors. However, they were also detected in activated plasmatocytes and could therefore be regarded as general indicators of hemocyte activation rather than specific markers of lamellocytes. The same could be said for the *msnCherry in vivo* marker.

## Discussion

**Fig 11:**
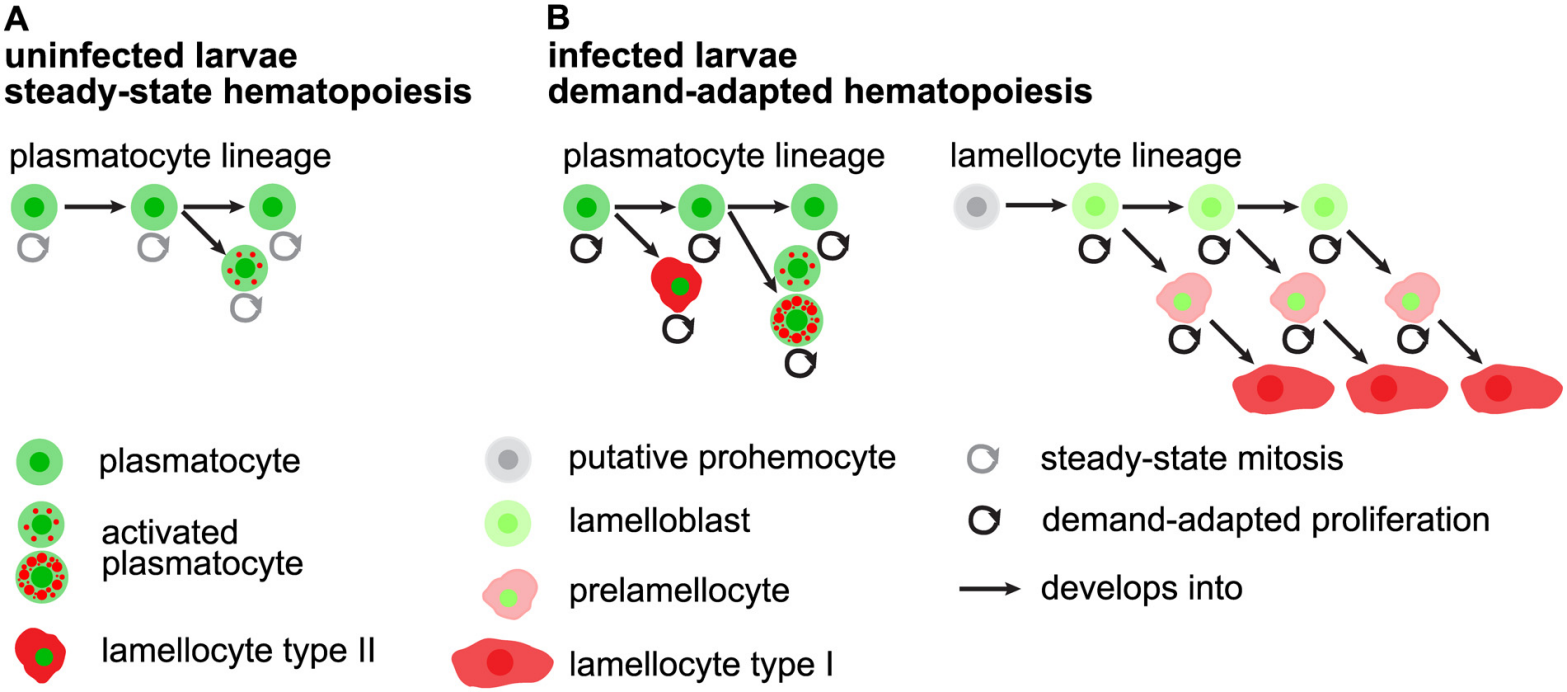
Model. (A) Steady-state hematopoiesis: Hemocytes of the plasmatocyte lineage prevail. Plasmatocytes self-renew, proliferate, and give rise to a small percentage of activated plasmatocytes. (B) Demand-adapted hematopoiesis. After wasp infection, hemocytes of the plasmatocyte lineage increase their proliferation rate. Plasmatocytes give rise to activated plasmatocytes with large and abundant mCherry-positive foci. And plasmatocytes transdifferentiate into type II lamellocytes on the wasp egg. A wasp infection induces the lamellocytes lineage. Putative prohemocytes that reside within the sessile hemocyte bands, and which are double-negative for the dual reporter constructs, give rise to circulating vigorously dividing lamelloblasts that transiently express GFP. These cells develop into type I lamellocytes via an intermediate prelamellocyte. All cell types are able to divide with the exception of type I lamellocytes.

The switch from steady-state to infection-induced hematopoiesis in *Drosophila melanogaster* is marked by the generation of a new blood cell type, the lamellocyte. Three different models for lamellocyte hematopoiesis have been proposed. Firstly, prohemocytes in the lymph glands self-renew and directly transform into lamellocytes that are released into the circulation [15,16,51]. Secondly, prohemocytes from the lymph glands [52], or putative prohemocytes in the circulation [20], develop into plasmatocytes, which transdifferentiate via so-called podocytes into lamellocytes [20,52]. Thirdly, peripheral plasmatocytes of procephalic origin transdifferentiate directly into lamellocytes [21–23]. Nonetheless, the dynamics of lamellocyte hematopoiesis remain largely elusive. Here we present a two-lineage model for lamellocyte hematopoiesis, where one type of lamellocytes is generated from the plasmatocyte lineage, by direct transdifferentiation of plasmatocytes on the surface of the parasite, and the other from a designated lamellocyte lineage, with infection-induced lamelloblasts that differentiate into circulating lamellocytes (Fig 11).

Lamelloblasts are characterized by the expression of the plasmatocyte markers *eaterGFP* and NimC1, albeit the *eaterGFP* expression level is ten times lower in lamelloblasts than in plasmatocytes. Therefore, we first assumed that lamelloblasts might be generated from plasmatocytes by downregulating *eaterGFP* or via the cell division of plasmatocytes, which would dilute the GFP fluorescence. But several arguments speak against these ideas. Firstly, as GFP is a highly stable molecule with a half-life of 24 hours or more [53,54], it seems unlikely that downregulation would result in such a large difference in GFP expression after just one round of cell division. Secondly, lamelloblasts appear suddenly 8 h after infection, without the presence of obvious precursors among the circulating cells. The lamelloblast count increased from zero to more than 1000 cells in only six hours, whereas plasmatocyte numbers remained at a constant level. Producing this many lamelloblasts in one cell division would entirely deplete the plasmatocyte pool. Asymmetric cell division could in principle have generated cells with reduced levels of GFP expression, but we never observed mitotic plasmatocytes with unequal distribution of nuclear GFP expression. Furthermore, lamelloblasts are a uniform population of small cells with lower granularity than observed in plasmatocytes. Several other studies attribute similar features to prohemocytes [15,20,21,52]. Taken together, these features establish lamelloblasts as a population that is clearly distinct from plasmatocytes and suggest a non-plasmatocyte origin for these cells.

Several of our findings imply that lamelloblasts derive directly from sessile prohemocytes. We showed that knocking down the cytokine Edin in the fat body reduced the number of lamelloblasts in the circulation. Furthermore, we previously found that sessile cells are not released into the circulation in response to a wasp infection in *edin* knockdown larvae [48]. This indicates that the precursor cells of lamelloblasts likely reside in the sessile tissue. In addition, the Eater protein was originally described as a phagocytosis receptor on plasmatocytes [55], but recently it was shown that it is also required for the attachment of hemocytes to the sessile compartment [56]. This might suggest that the low expression level of *eaterGFP* in lamelloblasts induces their release from the sessile islets. Márkus et al. [18] found that cells expressing neither plasmatocyte nor lamellocyte antigens were lost from the sessile population after a wasp infection and that transplanting sessile hemocytes into recipient larvae triggered lamellocyte hematopoiesis in the transplanted cells. This shows that sessile hemocytes can be a source of lamellocytes.

The relative contribution of lymph glands to circulating hemocytes during the immune response is still uncertain. Lymph glands release prohemocytes, plasmatocytes, and lamellocytes into the circulation [21], but only after the immune response against the wasp egg has already started [18]. Furthermore, we show that even though the primary lymph gland lobes stay intact after a *L*. *heterotoma* infection, all hemocyte types of the lamellocyte lineage develop normally. Still, the lymph glands likely contribute to the population of lamellocytes or their precursors at later time points after an infection.

We confirmed that lamellocytes are terminally differentiated and non-mitotic [15,20,47], but nevertheless we detected EdU-positive lamellocytes after a wasp infection. Biosynthetically active cell types are known to undergo endocycles characterized by the uncoupling of DNA-replication from mitosis. Endoreplicating cells typically go through the S- and G1-phases of the cell cycle [57]. Therefore EdU-incorporation in lamellocytes could be due to endoreplication, but the absence of *>S/G2/M-Green* expression in lamellocytes indicates that DNA-synthesis is quiescent. Although we cannot entirely exclude that lamellocytes endoreplicate, we suggest that they originate from mitotically active precursor cells, namely lamelloblasts and prelamellocytes.

The plasmatocyte lineage originates from procephalic plasmatocytes. At the time point when the wasp eggs hatch, activated plasmatocytes appear in large numbers. This suggests that plasmatocytes play an important role in the defense response, although we did not see them on the wasp egg. When plasmatocytes are activated they become more granular, grow in size, and accumulate cytoplasmic mCherry-positive foci. The granular mCherry fluorescence in activated plasmatocytes may indicate the phagocytosis of lamellocyte-derived material, but the expression of lamellocyte antigens might also signify the general activation of the immune system.

An important question is how the hemocyte classes in *Drosophila* can be homologized to the blood cells of other species. Unfortunately, the naming of *Drosophila* hemocytes is not congruent with the generally accepted terminology for other insect orders, including other dipterans. *Drosophila* plasmatocytes are structurally and functionally very similar to the professional phagocytes of other insects, usually called granulocytes or granular cells, and these cell types are considered homologous [3,58]. On the other hand, general insect terminology reserves the term plasmatocyte for a granulocyte-like, but agranular, class of cells that actively participate in the encapsulation of parasites, much like the *Drosophila* lamellocytes [3,58]. Our observation that *Drosophila* lamellocytes actually originate from a group of round cells of low granularity, the lamelloblasts, suggests that the lamellocyte lineage may indeed be homologous to the plasmatocytes of other insects. Notably, hemocytes of lamellocyte morphology are only found among the *Drosophila* species of the *melanogaster* subgroup [59], although lamellocyte-like cells have been described in several other drosophilids [59,60]. Instead of lamellocytes, some drosophilids have evolved other bizarre hemocyte types that participate in the encapsulation of parasitoid wasps, such as the hairy pseudopodocytes of the *obscura* group [61] and the highly motile multinucleated giant hemocytes of the *ananassae* subgroup [62]. In the drosophilid *Zaprionus indianus* and several *Drosophila* species, spindle- or thread-shaped nematocytes appear together with lamellocyte-like cells [60]. A parsimonious interpretation of these observations is that an ancestral hemocyte class, specialized in the encapsulation of parasites, has undergone rapid and diversifying evolution in the *Drosophila* lineage.

Homologies between *Drosophila* and human blood cells remain entirely speculative. Indeed, it is not unlikely that different types of blood cells evolved independently in vertebrates and arthropods. Still, the activation of plasmatocytes in the plasmatocyte lineage can be seen as an interesting analogy to the transformation of monocytes into macrophages.

Peripheral plasmatocytes proliferate by self-renewal [19] and their numbers increase during larval development [20,49]. Hemocyte numbers have also been shown to increase after a wasp infection [20,63,64]. This increase has been linked to the release of cells from the sessile compartment [17,18] and from the lymph glands [3,15,21]. Our results show that the demand-adapted hematopoiesis of the lamellocyte and plasmatocyte lineages is reason for the increase in cell counts after a wasp infection. Moreover, hemocytes divide on the wasp egg and in the sessile compartment. Infection-induced mitosis has been observed in *Anopheles gambiae* [65,66], but to our knowledge this has not previously been demonstrated for immune-induced cell types in *Drosophila*. In mammals, on the other hand, demand-adapted hematopoiesis is a well described trait of the immune response and is characterized by the increase of cell numbers several-fold over the steady-state levels of blood cell production [67–69]. Recently, subpopulations of tissue macrophages derived from embryonic cells were also found to divide *in situ* rather than being replenished by myelopoiesis [70,71]. Taken together, the immune response after wasp infection is reminiscent of the demand-adapted hematopoiesis in mammals.

Antibodies have been instrumental in defining blood cell populations in *Drosophila* larvae, where the expression of the P1/NimC1 antigen marks plasmatocyte identity and the expression of the L-antigens lamellocyte identity [28]. *eaterGFP* and *msnCherry* have been introduced as specific markers for plasmatocytes and lamellocytes respectively [30,32]. However, after immune activation, cells with plasmatocyte morphology express varying levels of L-antigens, indicating that they represent intermediate cell types [4,21]. Similarly, we show that the expression of *eaterGFP* and *msnCherry* is not restricted to plasmatocytes or lamellocytes, but that cell populations expressing both reporter constructs exist. Available plasmatocyte and lamellocyte markers unambiguously define unchallenged plasmatocytes and fully differentiated lamellocytes, respectively, but because of the dynamic nature of the immune response a combination of reporter constructs, or the corresponding plasmatocyte and lamellocyte antibodies, have to be used in order to define the blood cell lineages.

In conclusion, flow cytometry in combination with fluorescent hemocyte markers is an accurate and fast method for the differential counting of *Drosophila* blood cell populations from single larvae, and is potentially useful for the high throughput analysis of hemocyte phenotypes in genetic screening, or drug testing *in vivo*. Overall, our findings show that lamellocytes are generated in parallel by the transdifferentiation of plasmatocytes and *de novo* from lamelloblasts. However, the origin of lamelloblasts remains uncertain. The challenge is now to create appropriate genetic tools to track and experimentally manipulate individual hemocyte populations and to understand how the as yet elusive signals from different tissues, like the fat body and somatic muscles [44,48,72], integrate to shape a functional immune response.

## Materials and Methods

### Wasp and fly husbandry and genetics

We maintained the wasp strains *Leptopilina boulardi G486* (*L*. *boulardi*), *Leptopilina heterotoma strain 14* (*L*. *heterotoma*) on *D*. *melanogaster Canton S*, and *Leptopilina clavipes* (*L*. *clavipes*) on *D*. *virilis*. Wasps were collected into vials containing apple juice agar. All fly cultures were maintained on mash potato food.

We generated a double reporter line, which we refer to as *Me*, by meiotically recombining the X chromosome-linked *eaterGFP* with a nuclear localization signal and cytoplasmic *MSNF9MOmCherry* (*msnCherry*) constructs. Crosses of *Me* with *w* ^*1118*^ *iso* (*Me*/*w*) were used for most experiments. We recombined the second-chromosome-linked *eaterDsRed* and *Hml* ^*Δ*^-*GAL4* (*Hml* ^*Δ*^>) constructs and combined these flies with *msnCherry* to create *msnCherry*;*eaterDsRed*,*Hml* ^*Δ*^>. In addition, we recombined the third-chromosome-linked *UAS*-*S*/*G2*/*M*-*Green* with *He*-*GAL4* (*He*>) to create *w*;*He*>*S*/*G2*/*M*-*Green*. For the *edin* experiments, we used the fly lines published in [48]. The hemocyte double reporter was always homozygous for the *edin RNAi* and its control (*Me*;*Fb*-*GAL4* crossed to *Me*;*edinKK* ^*109528*^ and *Me* crossed to *Me*) and heterozygous for *UAS*-*edin* (*Me*;*Fb*-*GAL4* crossed to *UAS*-*edin* and *Me* crossed with *UAS*-*edin* as control). All wasp species and fly stocks used in this study as well as their origins are summarized in S1 and S2 Tables. For all experiments, we crossed 20 female and ten male flies. When the fly cultures started to produce eggs, the flies were tipped daily and the vials containing eggs were moved to a 25 or 29°C temperature controlled incubator, whereas the parental crosses were kept at room temperature.

### Melanization and killing of wasp larvae

On the third day after egg laying, we added twenty female and male wasps to the fly larvae cultures and allowed the wasps to infect fly larvae at room temperature for 2 h, removed the wasps and placed the fly vials back into 25 or 29°C.

In order to determine whether the cellular immune system of *Drosophila* larvae had mounted an encapsulation response, we dissected larvae 27–29 h after infection in water on twelve well slides and scored the degree of encapsulation according to the following phenotypes: *type 1*: no melanin deposit on the wasp egg; *type 2*: melanin deposit on the wasp egg on less than 50% of wasp egg length; *type 3*: melanin deposit on wasp egg 50–99% of wasp egg length; *type 4*: wasp egg completely melanized and black. We pooled *type 2*, *3* and *4* and referred to them as infection type *melanized wasp eggs* and to *type 1* as infection type *non*-*melanized wasp eggs*.

Correspondingly, we investigated if the larval immune system had killed wasp larvae 48–50 h after infection. We dissected fly larvae as described above and scored the phenotypes according to the following categories: *L* (living, non-melanized wasp larvae or eggs): living wasp larvae in the body cavity of the fly larvae, no melanization present; *LM* (living, melanized)—living wasp larvae and remnants of melanized capsules or melanized wasp eggs or wasp larvae; *M* (dead, melanized)–killed and melanized wasp eggs or wasp. We grouped *L* and *LM* under the infection type category *living wasp larvae*, and *M* under *killed wasp larvae*.

We determined the encapsulation and killing ability of the *Drosophila* immune system with all three *Leptopilina* species. For the encapsulation as well as the killing experiments we scored 50 infected larvae of three independent crosses.

### Time line of a wasp infection

In order to detect changes in hemocyte types and numbers during the cellular immune response to a wasp infection on a narrow time scale, we analyzed hemocyte samples of at least ten individual *L*. *boulardi*-infected larvae of each available infection type and ten age-matched uninfected control larvae every second hour until 50 h after infection. Furthermore, we analyzed hemocyte samples of age-matched control and *L*. *boulardi*-infected larvae at 20, 30, and 48 h after infection raised at 25°C to control for temperature effects. Finally, we analyzed hemocyte samples of at least ten larvae infected by *L*. *heterotoma* and *L*. *clavipes* and age-matched uninfected control larvae 8, 12, 14, 18, 20, 22, 24, 28, 38, and 48 h after infection.

### Hemocyte sample preparation for flow cytometry and imaging

We collected larvae and washed them carefully in water with a brush until they were clean, dried them on tissue paper before we placed individual larvae into 20 μl sterile filtered (0.45 μm, ministart syringe filter) phosphate-buffered saline (PBS: 0.14 M NaCl, 0.0027 M KCl, 0.01 M Phosphate pH 7.4) with 8% bovine serum albumin (BSA, Sigma) on 12-well slides (Thermo Scientific). Then, we dissected the larvae with biology tip forceps (Fine Science Tools), careful not to puncture the intestinal tract, gently shaking the carcasses to bring more lose blood cells into suspension. At this point, the infection status of the larvae was visually inspected. We removed the carcasses and pipetted the hemocyte samples into earlier prepared tubes containing 80 μl PBS with 8% BSA and analyzed the samples with a flow cytometer. For imaging, the blood cells remained on the 12-well slides and were allowed to spread in a humidified chamber for one hour.

### Microscopy

We used a Zeiss LSM 780 laser scanning confocal microscope mounted on a Cell Observer microscope for imaging DAPI (pulsed diode laser 405 nm), GFP (multiline Argon laser 488 nm), DsRed (multiline Argon laser 514 nm), mCherry (HeNE-laser 594 nm or diode laser 561 nm), Alexa Fluor 647 (*InTune*-tunable pulsed laser 628 nm), and Alexa Fluor 680 (*InTune*-tunable pulsed laser 628 nm). A Zeiss AxioImager M2 equipped with an ApoTome 2 and COLIBRI LED was used to image Alexa Fluor 405 (LED 380 nm), GFP (LED 470 nm), mCherry (LED 555 nm), and DIC with an AxioCam HRm CCD camera.

### Flow cytometry

A BD Accuri C6 flow cytometer was used to analyze cell numbers and cell types. We analyzed 30 μl of the hemocyte suspension. GFP-positive cells were recorded by the FL1 detector equipped with a 510/15 BP filter and mCherry-positive cells by the FL3 detector with a 610/20 BP filter. Both channels were excited using a 488 nm blue laser. In order to control fluorescence spill over, we pooled hemocytes of three to five late L3 *w* larvae as a negative control, hemocytes of *eaterGFP* females crossed to *w* males as a GFP-only control, and hemocytes of wasp-infected larvae (48 h *pi*) of a cross of *msnCherry* females to *w* males as a mCherry-only control. We corrected the spillover of GFP into the FL3 detector by subtracting 8% of FL1. mCherry did not spill into FL1. A description of the gating strategy can be found in S2 Fig.

We used hemocytes of ten pooled late L3 *w* larvae that were previously fed with EdU and had undergone the Click-iT Plus EdU Flow Cytometry Assay protocol with Alexa Fluor 647 picolyl azide as Alexa^647^-only control. EdU-Alexa^647^ was detected by FL4 equipped with a 675/25 filter. We corrected the spillover of Alexa^647^ by subtracting 1.61% of FL3. For a detailed description of the gating strategy see S9 Fig.

To estimate hemocyte size, we used a standard kit of Polystyrene particles with the diameters of 2.1 μm, 3.4 μm, 5.1 μm, 7.4 μm, 10.5 μm, and 14.7 μm (Spherotech Inc.). We diluted the particles according to the manufacturer’s instructions in PBS and ran 5000 events per bead size.

### Cell sorting

A BD FACSAria II instrument with the FACSDiva v8.0 software was employed to sort the different fluorescent hemocyte populations. Hemocytes of *w* were used as a negative control, hemocytes of infected *msnCherry*/*w* as an mCherry-only control, and hemocytes of uninfected *Me*/*w* served as the GFP-only control to set the gates for cell sorting. Hemocytes of *Me*/*w* infected by *L*. *boulardi* and *L*. *clavipes* collected 28 h *pi* were used to retrieve all hemocyte populations. Yield was favored over specificity in the sorting process. After sorting, the cells were pipetted onto 12-well slides to spread for 1 h in a humidified chamber and treated accordingly to the immunohistochemistry protocol (see below). Finally the cells were mounted with ProLong Gold Antifade Mountant with DAPI (Life Technologies) and imaged with a Zeiss LSM 780 confocal microscope.

### Imaging of whole mount *Drosophila* larvae, wasp eggs, and wasp larvae

We used uninfected and infected *Me*/w larvae, carefully cleaned them in water with a brush, and mounted them with their dorsal side facing upwards in a drop of ice-cold 100% glycerol on a glass slide. Double-sided sticky tape was used to attach the cover slip tightly onto the larvae. *MsnCherry*/*w*; *Hml* ^*Δ*^>*GFP*/*+* larvae were used instead of *Me/w* larvae at early time points due to low fluorescence of the *Me* reporter in whole larval mounts. The mounted larvae were kept at -20°C for 20 minutes or at 4°C for four to eight hours prior to imaging in order to make sure the larvae were not moving during the imaging. Larvae were imaged with a Zeiss Apotome.2 microscope with an EC Plan Neofluar 5x/0.16 objective (pixel size 6.45 μm^2^) or with an EC Plan Neofluar 10x/0.30 objective (pixel size 6.45 μm^2^). We processed all whole mount larval images with and without structured illumination to ascertain the presence of lymph glands. Primary lymph gland lobes are often covered by the fat body. Optical sectioning causes imaging artifacts that result in erasing lymph gland lobes in the apotomized image. We therefore determined the presence of primary lymph gland lobes by analyzing the raw data Z stacks. We dissected out wasp eggs at several time points after infection, fixed them in 4% paraformaldehyde, washed and mounted them in 50% glycerol in PBS. Then wasp eggs were imaged with a Zeiss Apotome.2 microscope with an EC Plan Neofluar 20x/0.50 objective (pixel size 12.90 μm^2^).

### EdU incorporation experiments for flow cytometry and imaging

We used the Click-iT Plus EdU Alexa Flour 647 Flow Cytometry Assay Kit and the Click-iT EdU Alexa Fluor 647 Imaging Kit (Life technologies) to mark hemocytes in the S-Phase of the cell cycle. The preparation of EdU-containing food was identical for imaging and flow cytometry. We melted the fly food, added red food coloring, and let the food cool down to below 70°C before adding EdU with a final concentration of 0.2 mM. When the food had cooled down entirely, age-matched control and infected larvae were allowed to feed on it for 4 or 12 h at room temperature. Feeding of EdU food was ensued by the following incubation schemes on normal food until the larvae were dissected. Feeding from 2–6 h, 4–8 h, and 2–12 h *pi* and analyses at 8–10 h, 8–10 h, and 12–14 h *pi*, respectively, answered the question, which cell types divided at early time points. Feeding from 4–8 h and 2–12 h and analyses at 28–30 h *pi* helped to find out if any population would give rise to lamellocytes. And finally, feeding from 28–32 h *pi* and analysis at 48 h *pi* shed light on whether activated plasmatocytes and lamellocytes divided. Only larvae with red food coloring in their intestinal tract were dissected to ensure that they had consumed EdU.

For imaging, we pooled hemocytes of two larvae per well. The cells were treated and mounted as described above. Instead of adding the primary antibody, we proceeded according to the protocol of the manufacturer’s instructions with the following exception: We reduced the amount of the reaction cocktail to 1/8 of the suggested amount.

For flow cytometry, 40 to 60 larvae were dissected for the early feeding and dissection schemes and about 20 for the late. We proceeded according to the manufactures instruction with the following exceptions: We pooled hemocytes and centrifuged them at 2500 x g for 5 min. We washed the cells with 500 ul of 1% BSA in PBS. We used 50 μl of Click-iT fixative. We re-suspended the cells in 100 μl of Click-iT saponin-based permeabilization and wash reagent, and analyzed the cells by flow cytometry as described previously.

### Time lapse study of blood cells on wasp eggs

In order to further investigate the development of lamellocytes type II on wasp eggs, we infected *Me*/w by *L*. *boulardi*, dissected out the eggs 16 h after infection, and imaged them every 25 min for 14 h to record changes in hemocyte populations. Glass bottom culture dishes (MatTek) were coated with 1% Gelatine (Sigma) in PBS for one hour. The culture medium was Schneider´s medium (Sigma) with 25% FBS (Sigma) [73]. The eggs were carefully placed into glass bottom dishes and allowed to settle for 30 min before they were imaged with a Zeiss LSM 780 confocal microscope using a 20x/0.8 Apochromat objective equipped with DIC M27. 3D stacks with 1 μm slice intervals were acquired every 25 min for 14 h. The time lapse series was then processed with ImageJ and iMorph to assemble a time lapse movie.

### Immunohistochemistry and counting the proportions of cell types stained

NimC1/P1, Atilla/L1, L2, L4 and L6 hemocyte-specific antibodies [28] were used to stain hemocytes of five to six individual infected and age-matched uninfected larvae at 8, 18, 20, 28 and 48 h after infection. The infections and dissections of the larvae were done as described above, and each antibody was applied on a separate set of samples. Dissected hemocytes were allowed to spread on glass slides in humidified chambers for approximately 1 h. The cells were fixed with ice-cold 4% paraformaldehyde in PBS for 10 min and washed three times with ice-cold PBS, followed by permeabilization with 0.1% Triton X-100 for 5 min. After washing three times with PBS, cells were blocked with 3% BSA in PBS for 1 h at room temperature. After removing the blocking solution, 20 μl of the primary antibody supernatant was added for an overnight incubation at 4°C. Cells were again washed three times with PBS and incubated with a goat anti-mouse Alexa Fluor 680 secondary antibody (Life Technologies) in 1% BSA in PBS (1:500) for 1 h at room temperature in the dark. Finally, the cells were washed and mounted with ProLong Gold Antifade Mountant with DAPI (Life Technologies) and high precision cover glasses (Zeiss). The cells were imaged using a Zeiss LSM 780 confocal microscope with a Plan Apochromat 63 x/1.4 oil immersion objective. The images were processed using ImageJ. The negative controls (either missing primary or secondary antibody) were used to set a threshold level for positive antibody staining. On each image, we manually classified the cells into five different subtypes based on their GFP and mCherry expression and morphology as described above (Fig 1). Then we calculated the ratio of cells stained with a specific antibody to total number of that cell type at each time point. The amount of different cell types based on *eaterGFP* and *msnCherry* expression varied considerably among the time points after infection (Fig 5). The total number of cells analyzed per antibody per time point was between 69 and 256 (generally over 100), except for the 8–10 h time point, where the cell numbers were generally low, around 20. Antibody—time point—cell type combinations with less than ten cells were excluded from the analysis.

Wasp eggs were dissected 14–16 h after infection by *L*. *boulardi* in PBS. Eggs were stained with NimC1/P1, L4, and Atilla/L1 antibody as described for antibody staining of bled hemocytes. Alexa Fluor 405 was used as the secondary antibody. Wasp eggs were imaged with a Zeiss Apotome.2 microscope with a EC Plan Neofluar 20x/0.5 objective (pixel size 12.90 μm^2^).

### Statistical analysis and plotting

For all statistical analysis we used R (version 3.1.3 (2015-03-09)—"Smooth Sidewalk" Copyright (C) 2015 The R Foundation for Statistical Computing). We used statistics to find out whether the infection type affected the total number of blood cells. The only time points after *L*. *boulardi* infection when more than one infection type was present were 36 to 50 h after infection, and for *L*. *clavipes* 24 h after infection. We also wanted to know if edin-knock down affected total number of blood cells and the number of individual blood cell types. Finally, we compared the size and granularity of the different hemocyte types.

First, we tested the data for normality and homoscedasticity. When these requirements were met, we used two-way ANOVA with an interaction term followed by Tukey´s HSD test. If the requirements were not met regardless of transformation, we used the non-parametric Kruskal-Wallis rank sum test and the independent two-group Mann-Whitney U Test for pairwise testing. In the *edin* experiments our data contained two or three independent replicates for each genotype. Therefore, we separately tested if replicates and if genotypes differed significantly. Then we made pairwise comparisons between *edin* knockdown and overexpression to their respective controls. For plotting we used ggplot2 [74].

## Supporting Information

S1 Fig Melanization and killing of wasp eggs and larvae. (A) Proportion of *Drosophila* larvae with melanized (27–29 h after infection) or killed (48–50 h after infection) *L*. *boulardi*, *L*. *clavipes*, and *L*. *heterotoma* eggs or larvae. Three independent experiments of at least 50 infected *Me/w* heterozygous *Drosophila* larvae are shown. (B-B”“) Representative images of non-melanized (B) and (B’-B”“) melanized wasp eggs. The melanization pattern of *L*. *boulardi* and *L*. *clavipes* eggs was different. *L*. *boulardi* eggs were melanized up to 50% of the length of the wasp egg (B-B”), whereas *L*. *clavipes* eggs were melanized to between 75–100% of the length (B”‘-B”“). *L*. *heterotoma* eggs were never melanized nor encapsulated (B). Representative images of (C-C’) living and (C”) killed wasp larvae. (C’) *L*. *boulardi* larvae evaded the melanized capsule, (C”) but some of the wasp larvae were subsequently killed by the immune system. (C) *L*. *clavipes* eggs were readily melanized and encapsulated and killed wasp larvae were rarely observed. *L*. *heterotoma* larvae were rarely killed and living wasp larvae were present in the hemocoel. Scale bars 50 μm.
(PDF)

S2 Fig Gating strategy for the dual hemocyte reporter system *msnCherry*,*eaterGFP*. (A) Live gate. Hemocytes were easily distinguishable from debris when FSC-A was plotted against SSC-A on a logarithmic scale in a dot plot. All further analyses were applied to the live gate of hemocytes (red dashed ellipsoid). (B) Overlay histogram and (B’) scatterplot of hemocytes of w/w (black lines and black arrow) and *eaterGFP/w* (green line, green arrow, green dots) uninfected third instar larvae. (C) Overlay histogram and (C’) scatterplot of hemocytes of *w/w* (black lines and black arrow) and *msnCherry/w* (red line, black, yellow and red arrows, grey, yellow and red dots) third instar larvae 48 h after *L*. *boulardi* infection. The dashed blue lines mark the fluorescent intensities that were used to separate cell populations. GFP and mCherry were excited with a 488 nm solid state laser. GFP was detected by the FL1 detector equipped with a 510/15 BP filter and mCherry by the FL3 detector with a 610/20 BP filter. Non-fluorescent (*w/w*), GFP-only (*eaterGFP*/*w*), and mCherry-only (*msnCherry*/*w*) blood cells were used to set-up the flow cytometry experiments to simultaneously detect GFP and mCherry expressing (*Me*/*w*) cells. Fluorescent spillover of GFP into the FL3 detector was compensated by subtracting 8% from FL1. Hemocytes from *w/w* larvae were autofluorescent. These cells were used to set the threshold between non-fluorescent and fluorescent hemocyte populations (black lines and black arrows in B and C). Hemocytes of larvae of *eaterGFP*/*w* crosses had one peak with a high fluorescence intensity (green arrow, green line in B and green dots in B’). These cells represented the plasmatocyte population. The expression of mCherry was induced by a wasp infection. Hence hemocytes of third instar larvae of *msnCherry*/*w* had three fluorescent peaks: one with low fluorescent intensity (red line, black arrow in C and gray dots in C’), a second with intermediate fluorescent intensity (red line, yellow arrow in C and yellow dots in C’), and a third with high fluorescent intensity (red line, red arrow in C and red dots in C’). The left peak corresponded to the negative cell population that was comprised mainly of plasmatocytes, the center peak to double positive hemocytes consisting of activated plasmatocytes, lamellocytes type II and prelamellocytes, and the right peak to lamellocytes.
(PDF)

S3 Fig Images of hemocyte populations after cell sorting. (A-A”‘) plasmatocytes, (B-B”‘) lamelloblasts, (C-C”‘) activated plasmatocytes and lamellocytes type II, (D-D”‘) prelamellocytes, and (E-E‴) lamellocytes type I. All fluorescent channels and the merge are shown separately. Scale bars 10 μm.
(PDF)

S4 Fig Comparison of GFP intensity, granularity, and size of plasmatocytes, lamelloblasts, and activated plasmatocytes in *L*. *boulardi*-infected and age-matched wild type larvae. Values were acquired by the AccuriC6 flow cytometer. (A-A’) The GFP intensity of plasmatocytes after infection is ten-fold higher than that of lamelloblasts. Fluorescent intensity values were taken 14–16 h after infection when lamelloblasts were most abundant. (B) Distribution of size beads on the FSC-A axis. (C-C’) Plasmatocytes are more granular (SSC-A, C) and bigger (FSC-A, C’) than lamelloblasts. Values were taken 14–16 h after infection. (D-D’) After infection, plasmatocytes became less granular (SSC-A, C) and were smaller (FSC-A, C’) than the plasmatocytes of uninfected and activated plasmatocytes of infected larvae. Values were taken 48–50 h after infection. (E-E”) Size and granularity of blood cells. We first gated all blood cell populations according to the gating strategy described above and then backgated the blood cell populations onto the live gate P1. Hemocyte size and granularity increased when the cells advanced in the plasmatocyte and lamellocyte lineages. A scatter plot of blood cells of all lineages (E), a scatter plot of hemocytes of the plasmatocyte lineage (E’), and a scatter plot of hemocytes of the lamellocyte lineage (E”).
(PDF)

S5 Fig Timeline of total circulating hemocyte counts of control and wasp-infected larvae. (A) Total hemocyte counts of uninfected *Me*/*w* heterozygous larvae collected every second hour until 50 h. (B) Total counts after a *L*. *boulardi*, (C) a *L*. *clavipes*, and (D) a *L*. *heterotoma* infection. The box and whiskers plots depict the means of the total cell counts as red bars, the hinges of the box represent the upper and lower bound of the standard deviation (SD), and the whiskers reach to the lowest (Min) and highest (Max) measured cell number. Each dot represents the total cell count of an individual larva. In (B-D) the infection types are plotted as colored dots: Non-melanized wasp eggs as white and melanized wasp eggs as dark grey dots, living wasp larvae as light grey and killed wasp larvae as black dots. Blood cell numbers of at least ten age-matched control and *L*. *boulardi*-infected larvae were measured every second hour. The blood cells of larvae infected by *L*. *clavipes* and *L*. *heterotoma* were only counted at selected time points. Total blood cell numbers of control larvae increased slowly and rose suddenly at the two final time points (A). In *L*. *boulardi*-infected larvae, the total blood cell number suddenly increased 14 h after infection and stayed elevated (B). A similar pattern could be observed after a *L*. *clavipes* infection (C). However, total cell counts of *L*. *heterotoma*-infected larvae did not increase (D). The dynamics of total cell counts after *L*. *boulardi* and *L*. *clavipes* infections were comparatively equal, but the infection types were not. While eggs of *L*. *clavipes* started to melanize already at 22 h and were fully melanized 28 h after infection, the melanization of *L*. *boulardi* eggs was delayed. In fact, *L*. *boulardi* eggs only melanized very lightly and wasp larvae hatched around 30–32 h after infection. Wasp larvae of *L*. *clavipes* rarely hatched. The cellular immune system encapsulated the wasp eggs of *L clavipes*, but failed to encapsulate those of *L*. *boulardi*. Instead, *L*. *boulardi* larvae were attacked by blood cells and encapsulated. Eggs of *L*. *heterotoma* were never melanized or encapsulated, nor were the wasp larvae killed. The embryonic development of *L*. *heterotoma* seemed to take longer than for the other two species, as 38 h after infection wasp eggs of *L*. *heterotoma* could still be observed in the hemocoel, whereas *L*. *boulardi* had started to hatch already eight hours earlier. In order to test whether blood cell numbers depended on the type of infection, we applied Wilcoxon´s rank sum test to *L*. *boulardi* infections starting from 36 to 50 h (B, W = 6432.5, p = 0.933, n = 230) and Welch´s Two Sample T-test to *L*. *clavipes* 24 h after infection (C, T = 0.815, p = 0.425, n = 20). The blood cell numbers were not dependent on the infection type in either wasp species.
(PDF)

S6 Fig Circulating hemocyte counts of three time points of age-matched control as well as *L*. *boulardi*-infected larvae raised at 25°C. (A) Counts of all hemocyte classes of uninfected *Me/w* larvae (B-B’) and *L*. *boulardi*-infected larvae collected at the indicated time points. (C) Flow cytometry plots at three time points of *Me/w* and *L*. *boulardi*-infected larvae. Hemocytes of at least ten larvae were counted for each time point. The immune reactions of larvae at 25°C and 29°C were similar in the order of events. However, the immune reaction was slower due to the lower temperature.
(PDF)

S7 Fig Timeline of circulating hemocyte counts of age-matched control as well as *L*. *clavipes-* and *L*. *heterotoma*-infected larvae. (A) Counts of each hemocyte class of uninfected *Me*/*w* heterozygous larvae collected at the indicated time points, (B-B’) counts of *Me*/*w* heterozygous larvae infected by *L*. *clavipes*, and (C-C’) *L*. *heterotoma* hemocytes of at least ten individual larvae were counted. All cell types of *L*. *clavipes*–infected larvae followed similar kinetics as after a *L*. *boulardi* infection. After a *L*. *heterotoma* infection however, the count of all cell types stayed low. Lamelloblasts, prelamellocytes, and lamellocytes developed, but were eradicated by the *L*. *heterotoma* venom.
(PDF)

S8 Fig Images of circulating blood cells at representative time points after a wasp infection. (A-A”“) Uninfected *Me*/*w*-larvae, larvae of the same genotype infected by (B-B”“) *L*. *boulardi*, (C-C”“) *L*. *clavipes*, or (D-D”“) *L*. *heterotoma*. In uninfected larvae, plasmatocytes were the predominant blood cell type at all times. In *L*. *boulardi*- and *L*. *clavipes*-infected larvae, the first lamellocytes were seen in the circulation 18–20 h and 20–22 h after infection. At these time points, the mCherry expression was often very faint in comparison to later time points. *L*. *heterotoma*-infected larvae had only very few lamellocytes. Scale bars 50 μm.
(PDF)

S9 Fig Gating strategy for the dual hemocyte reporter system *Me* and EdU-Alexa^647^-labeled hemocytes. (A) Scatter plot of hemocytes and debris. Hemocytes were easily distinguishable from debris when FSC-A was plotted against SSC-A on a logarithmic scale. However, the Click.iT Plus EdU Flow Cytometry protocol led to an additional population of non-fluorescent cells with a lower forward scatter. We assumed that these cells had died in the process of detecting dividing cells, and we pointed them out as dead cells and therefore excluded them from the analysis. All further analyses were applied to the live gate of hemocytes (red dashed ellipsoid). (B) Overlay histogram of EdU-Alexa^647^-stained cells with non-fluorescent blood cells (black line—non-fluorescent cells; magenta line—EdU-Alexa^647^-positive cells). We used blood cells of ten pooled late L3 *w* larvae that were previously fed with EdU and had undergone the Click-iT Plus EdU Flow Cytometry protocol with Alexa Fluor 647 picolyl azide as EdU-Alexa^647^-only control, and *w* control larvae as negative control as described earlier. EdU-Alexa^647^ was excited with a 640 nm diode laser and detected with FL4 equipped with a 675/25 filter. We corrected the spillover of Alexa^647^ by subtracting 1.61% of FL3. (C) Overlay histograms of *eaterGFP*/*w* and (D) of *msnCherry*/*w* hemocytes that had undergone the entire Click-iT Plus EdU Flow Cytometry protocol without the click reaction (green line—GFP; red line—mCherry) and normally treated hemocytes according to our protocol (black lines). The Click-iT Plus technique is an improvement of the original Click-iT reaction, where copper catalyzes the covalent reaction between an alkyne in the ethynyl moiety of EdU and a picolyl azide coupled to Alex Fluor 647. The presence of copper and reactive oxygen species damages fluorescent molecules. Adding a copper chelate reduced the toxicity for fluorescent molecules (Life technologies). However, the reaction mix still reduced the fluorescent intensities of GFP and mCherry (black arrows in C and D). We adjusted the gates accordingly.
(PDF)

S10 Fig *In vivo* EdU incorporation of hemocytes after a wasp infection. (A-A’) Second instar *Me/w* larvae were infected by *L*. *boulardi* for two hours and subsequently placed on EdU-containing fly food for 12 h after a wasp infection. Hemocytes of these larvae were collected and stained immediately after feeding. The majority of hemocytes of infected larvae had incorporated EdU, indicating that they had been dividing. Hemocytes from control larvae had also divided at this time point. Stars in panel A’ mark an example of a lamelloblast and a plasmatocyte that incorporated EdU. (B-B’) EdU was fed to larvae 4–8 h after a wasp infection. Larvae were placed back on normal food and then dissected 28 h after infection. At this time point, the majority of prelamellocytes and lamellocytes type I were EdU-positive, suggesting that they originated from cells that had divided early after the infection. (C-C’) Larvae were feeding on normal fly food until 28 h after infection, after which they were put on EdU-food for four hours. Then, they were returned to normal food until they were dissected 48 h after infection. Then, only a small proportion of lamellocytes were EdU-positive, whereas other hemocyte types were EdU-positive at varying proportions, indicating that type I lamellocytes themselves were not dividing but all other cell types were. In B’ and C’ EdU-positive cells are marked with a star, examples of lamellocytes are marked with an arrow, activated plasmatocytes with filled arrowheads and prelamellocytes with open arrowheads.
(PDF)

S11 Fig Quantification of EdU results. (A-C) Quantification of different EdU experiments explained in the S10 Fig caption.
(PDF)

S12 Fig Visualization of hemocyte types with NimC1/P1, L4, and L6 hemocyte antibodies after a wasp infection. Second instar *Me*/*w* larvae were infected by *L*. *boulardi* for 2 h, hemocytes were collected at 8, 18, 20, 28, and 48 h after infection and stained with NimC1/P1 (first two panels), L4 (two middle panels) and L6 (two last panels) antibodies. For clarity, NimC1/P1, L4 and L6 stains are shown as layers separated from respective merged image. Hemocytes expressing GFP (plasmatocytes and lamelloblasts, activated plasmatocytes and prelamellocytes) were NimC1 positive after infection (A’-E’). Mature lamellocytes with no GFP expression were always NimC1 negative (B’-E’). Some, but not all, of the activated plasmatocytes were also stained with L4 (filled arrowheads, G’) as were prelamellocytes (G’) and mature lamellocytes (H’-J’). Some of the plasmatocytes were L4-postive already 8 hours after a wasp infection (F’), indicating their activation and/or transformation into lamellocytes. L6-positive hemocytes were detected at 18 h after infection (K-O). L6 mainly stained discoidal prelamellocytes (L-L’) and lamellocytes (L-O), but we also detected some L6-positive activated lamellocytes (N-N’). Arrows point to examples of lamellocytes, filled arrowheads to activated plasmatocytes and open arrowheads to prelamellocytes. “Greater than” signs (>) in M and M’ point to a lamellocyte type II that was stained with L6. Stars in D and D’ show a GFP and mCherry-negative, elongated cell that was stained with NimC1/P1 and in L and L’ a cluster of mCherry negative lamellocytes that were stained with L6. Size bars 10 μm.
(PDF)

S1 Table Wasp species used in the study. (PDF)

S2 Table Fly stocks used in the study. (PDF)

S1 Video Plasmatocytes transdifferentiated into type II lamellocytes. *L*. *boulardi* eggs were dissected 16 h after a wasp infection and cultured in a glass bottom dish. Hemocytes on the egg were imaged every 25 min for 14 h. GFP-positive plasmatocytes transdifferentiate into type II lamellocytes by increasing their expression of mCherry while retaining their GFP expression. White arrows point to two of several examples of plasmatocytes transdifferentiating into lamellocytes type II.
(MOV)
