# Supplementary material for: Transdifferentiation and Proliferation in Two Distinct Hemocyte Lineages in Drosophila melanogaster Larvae after Wasp Infection
Source: PLoS Pathog. 2016 Jul 14;12(7):e1005746. doi: 10.1371/journal.ppat.1005746 (PMC4945071; doi:10.1371/journal.ppat.1005746)
Supplement: S1 Fig — (A) Proportion of Drosophila larvae with melanized (27–29 h after infection) or killed (48–50 h after infection) L. boulardi, L. clavipes, and L. heterotoma eggs or larvae. Three independent experiments of at least 50 infected Me/w heterozygous Drosophila larvae are shown. (B-B”“) Representative images of non-melanized (B) and (B’-B”“) melanized wasp eggs. The melanization pattern of L. boulardi and L. clavipes eggs was different. L. boulardi eggs were melanized up to 50% of the length of the wasp egg (B-B”), whereas L. clavipes eggs were melanized to between 75–100% of the length (B”‘-B”“). L. heterotoma eggs were never melanized nor encapsulated (B). Representative images of (C-C’) living and (C”) killed wasp larvae. (C’) L. boulardi larvae evaded the melanized capsule, (C”) but some of the wasp larvae were subsequently killed by the immune system. (C) L. clavipes eggs were readily melanized and encapsulated and killed wasp larvae were rarely observed. L. heterotoma larvae were rarely killed and living wasp larvae were present in the hemocoel. Scale bars 50 μm. (PDF) [file ppat.1005746.s001.pdf]

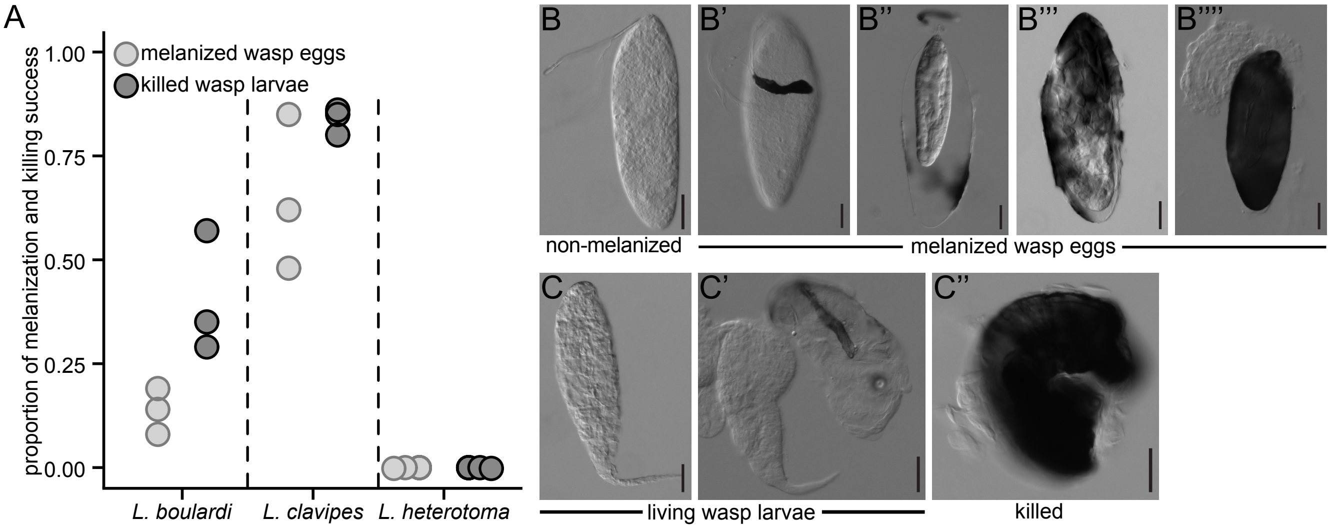

S1 Fig. Melanization and killing of wasp eggs and larvae. (A) Proportion of *Drosophila* larvae with melanized (27-29 h after infection) or killed (48-50 h after infection) *L. boulardi*, *L. clavipes*, and *L. heterotoma* eggs or larvae. Three independent experiments of at least 50 infected *Me/w* heterozygous *Drosophila* larvae are shown. (B-B'''') Representative images of non-melanized (B) and (B'-B'''') melanized wasp eggs. The melanization pattern of *L. boulardi* and *L. clavipes* eggs was different. *L. boulardi* eggs were melanized up to 50% of the length of the wasp egg (B-B''), whereas *L. clavipes* eggs were melanized to between 75-100 % of the length (B'''-B''''). *L. heterotoma* eggs were never melanized nor encapsulated (B). Representative images of (C-C') living and (C'') killed wasp larvae. (C') *L. boulardi* larvae evaded the melanized capsule, (C'') but some of the wasp larvae were subsequently killed by the immune system. (C) *L. clavipes* eggs were readily melanized and encapsulated and killed wasp larvae were rarely observed. *L. heterotoma* larvae were rarely killed and living wasp larvae were present in the hemocoel. Scale bars 50 μm.
