# Supplementary material for: Transdifferentiation and Proliferation in Two Distinct Hemocyte Lineages in Drosophila melanogaster Larvae after Wasp Infection
Source: PLoS Pathog. 2016 Jul 14;12(7):e1005746. doi: 10.1371/journal.ppat.1005746 (PMC4945071; doi:10.1371/journal.ppat.1005746)
Supplement: S2 Fig — (A) Live gate. Hemocytes were easily distinguishable from debris when FSC-A was plotted against SSC-A on a logarithmic scale in a dot plot. All further analyses were applied to the live gate of hemocytes (red dashed ellipsoid). (B) Overlay histogram and (B’) scatterplot of hemocytes of w/w (black lines and black arrow) and eaterGFP/w (green line, green arrow, green dots) uninfected third instar larvae. (C) Overlay histogram and (C’) scatterplot of hemocytes of w/w (black lines and black arrow) and msnCherry/w (red line, black, yellow and red arrows, grey, yellow and red dots) third instar larvae 48 h after L. boulardi infection. The dashed blue lines mark the fluorescent intensities that were used to separate cell populations. GFP and mCherry were excited with a 488 nm solid state laser. GFP was detected by the FL1 detector equipped with a 510/15 BP filter and mCherry by the FL3 detector with a 610/20 BP filter. Non-fluorescent (w/w), GFP-only (eaterGFP/w), and mCherry-only (msnCherry/w) blood cells were used to set-up the flow cytometry experiments to simultaneously detect GFP and mCherry expressing (Me/w) cells. Fluorescent spillover of GFP into the FL3 detector was compensated by subtracting 8% from FL1. Hemocytes from w/w larvae were autofluorescent. These cells were used to set the threshold between non-fluorescent and fluorescent hemocyte populations (black lines and black arrows in B and C). Hemocytes of larvae of eaterGFP/w crosses had one peak with a high fluorescence intensity (green arrow, green line in B and green dots in B’). These cells represented the plasmatocyte population. The expression of mCherry was induced by a wasp infection. Hence hemocytes of third instar larvae of msnCherry/w had three fluorescent peaks: one with low fluorescent intensity (red line, black arrow in C and gray dots in C’), a second with intermediate fluorescent intensity (red line, yellow arrow in C and yellow dots in C’), and a third with high fluorescent intensity (red line [file ppat.1005746.s002.pdf]

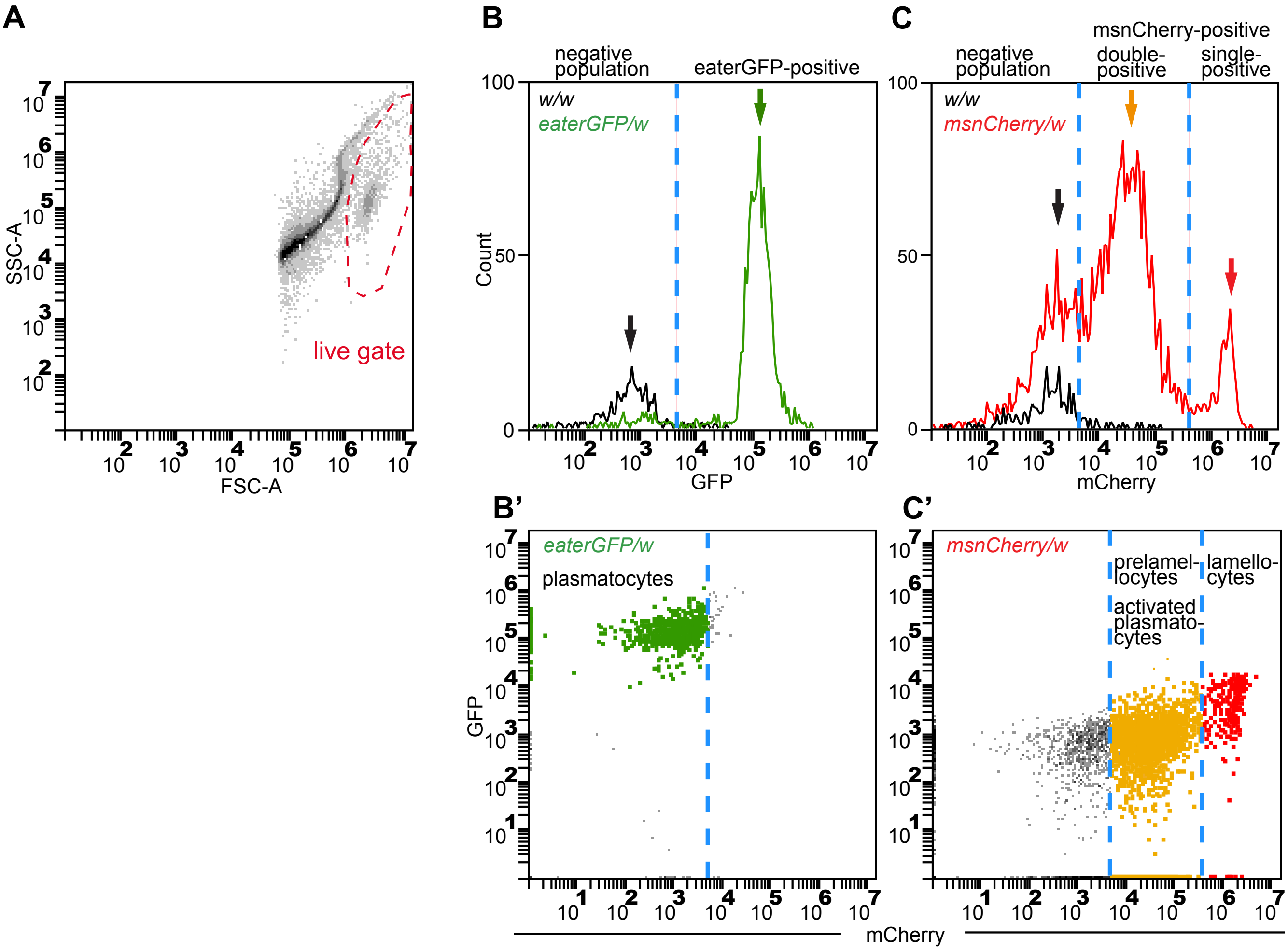

S2 Fig. Gating strategy for the dual hemocyte reporter system *msnCherry,eaterGFP*. (A) Live gate. Hemocytes were easily distinguishable from debris when FSC-A was plotted against SSC-A on a logarithmic scale in a dot plot. All further analyses were applied to the live gate of hemocytes (red dashed ellipsoid). (B) Overlay histogram and (B') scatterplot of hemocytes of w/w (black lines and black arrow) and *eaterGFP/w* (green line, green arrow, green dots) uninfected third instar larvae. (C) Overlay histogram and (C') scatterplot of hemocytes of *w/w* (black lines and black arrow) and *msnCherry/w* (red line, black, yellow and red arrows, grey, yellow and red dots) third instar larvae 48 h after *L. boulardi* infection. The dashed blue lines mark the fluorescent intensities that were used to separate cell populations. GFP and mCherry were excited with a 488 nm solid state laser. GFP was detected by the FL1 detector equipped with a 510/15 BP filter and mCherry by the FL3 detector with a 610/20 BP filter. Non-fluorescent (*w/w*), GFP-only (*eaterGFP*/*w*), and mCherry-only (*msnCherry*/*w*) blood cells were used to set-up the flow cytometry experiments to simultaneously detect GFP and mCherry expressing (*Me*/*w*) cells. Fluorescent spillover of GFP into the FL3 detector was compensated by subtracting 8 % from FL1. Hemocytes from *w/w* larvae were autofluorescent. These cells were used to set the threshold between non-fluorescent and fluorescent hemocyte populations (black lines and black arrows in B and C). Hemocytes of larvae of *eaterGFP*/*w* crosses had one peak with a high fluorescence intensity (green arrow, green line in B and green dots in B'). These cells represented the plasmatocyte population. The expression of mCherry was induced by a wasp infection. Hence hemocytes of third instar larvae of *msnCherry*/*w* had three fluorescent peaks: one with low fluorescent intensity (red line, black arrow in C and gray dots in C'), a second with intermediate fluorescent intensity (red line, yellow arrow in C and yellow dots in C'), and a third with high fluorescent intensity (red line, red arrow in C and red dots in C'). The left peak corresponded to the negative cell population that was comprised mainly of plasmatocytes, the center peak to double positive hemocytes consisting of activated plasmatocytes, lamellocytes type II and prelamellocytes, and the right peak to lamellocytes.
