# Supplementary material for: Transdifferentiation and Proliferation in Two Distinct Hemocyte Lineages in Drosophila melanogaster Larvae after Wasp Infection
Source: PLoS Pathog. 2016 Jul 14;12(7):e1005746. doi: 10.1371/journal.ppat.1005746 (PMC4945071; doi:10.1371/journal.ppat.1005746)
Supplement: S3 Fig — (A-A”‘) plasmatocytes, (B-B”‘) lamelloblasts, (C-C”‘) activated plasmatocytes and lamellocytes type II, (D-D”‘) prelamellocytes, and (E-E‴) lamellocytes type I. All fluorescent channels and the merge are shown separately. Scale bars 10 μm. (PDF) [file ppat.1005746.s003.pdf]

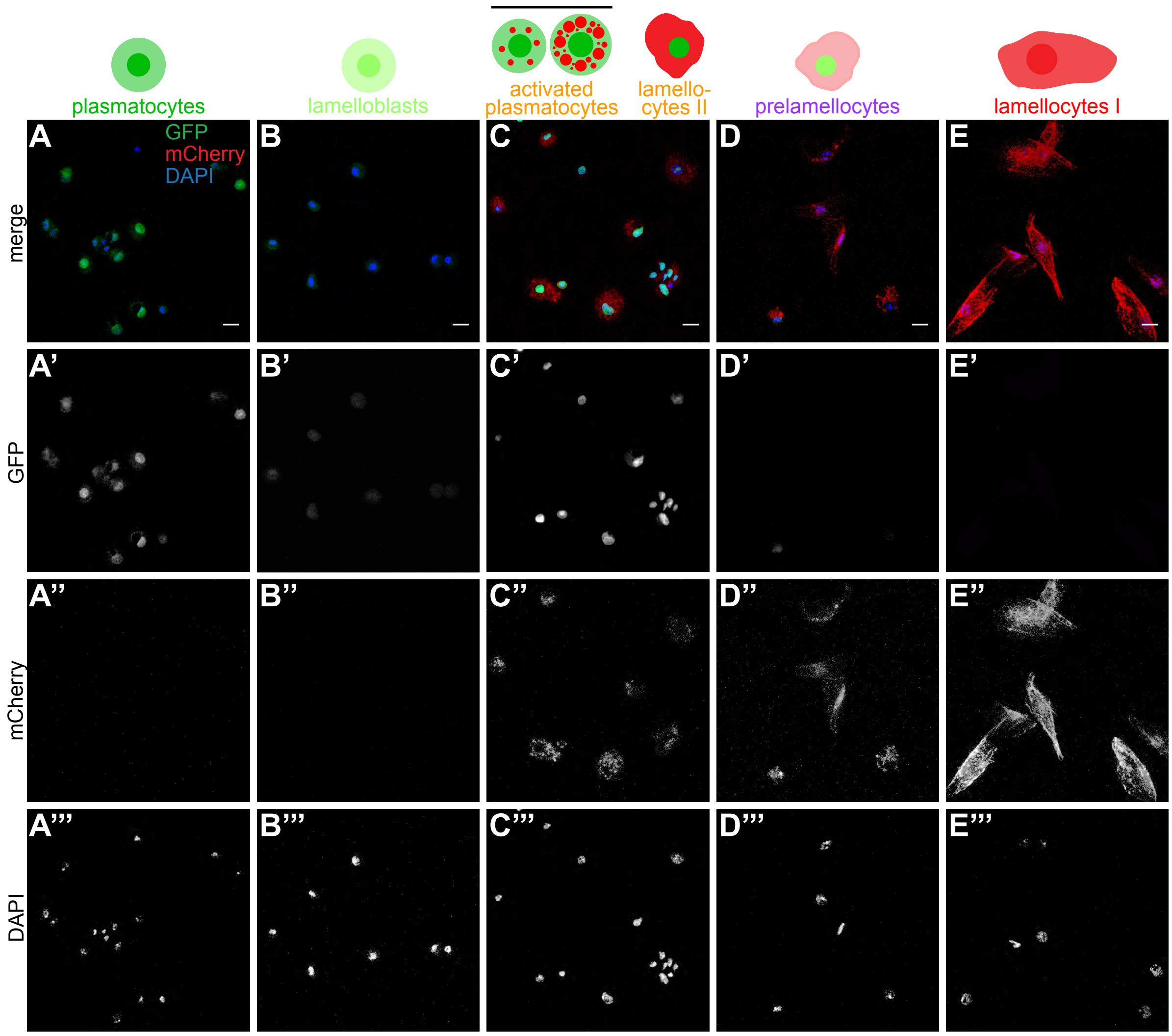

S3 Fig. Images of hemocyte populations after cell sorting. (A-A''') plasmatocytes, (B-B''') lamelloblasts, (C-C''') activated plasmatocytes and lamellocytes type II, (D-D''') prelamellocytes, and (E-E''') lamellocytes type I. All fluorescent channels and the merge are shown separately. Scale bars 10 μm.
