# Supplementary material for: Transdifferentiation and Proliferation in Two Distinct Hemocyte Lineages in Drosophila melanogaster Larvae after Wasp Infection
Source: PLoS Pathog. 2016 Jul 14;12(7):e1005746. doi: 10.1371/journal.ppat.1005746 (PMC4945071; doi:10.1371/journal.ppat.1005746)
Supplement: S4 Fig — Values were acquired by the AccuriC6 flow cytometer. (A-A’) The GFP intensity of plasmatocytes after infection is ten-fold higher than that of lamelloblasts. Fluorescent intensity values were taken 14–16 h after infection when lamelloblasts were most abundant. (B) Distribution of size beads on the FSC-A axis. (C-C’) Plasmatocytes are more granular (SSC-A, C) and bigger (FSC-A, C’) than lamelloblasts. Values were taken 14–16 h after infection. (D-D’) After infection, plasmatocytes became less granular (SSC-A, C) and were smaller (FSC-A, C’) than the plasmatocytes of uninfected and activated plasmatocytes of infected larvae. Values were taken 48–50 h after infection. (E-E”) Size and granularity of blood cells. We first gated all blood cell populations according to the gating strategy described above and then backgated the blood cell populations onto the live gate P1. Hemocyte size and granularity increased when the cells advanced in the plasmatocyte and lamellocyte lineages. A scatter plot of blood cells of all lineages (E), a scatter plot of hemocytes of the plasmatocyte lineage (E’), and a scatter plot of hemocytes of the lamellocyte lineage (E”). (PDF) [file ppat.1005746.s004.pdf]

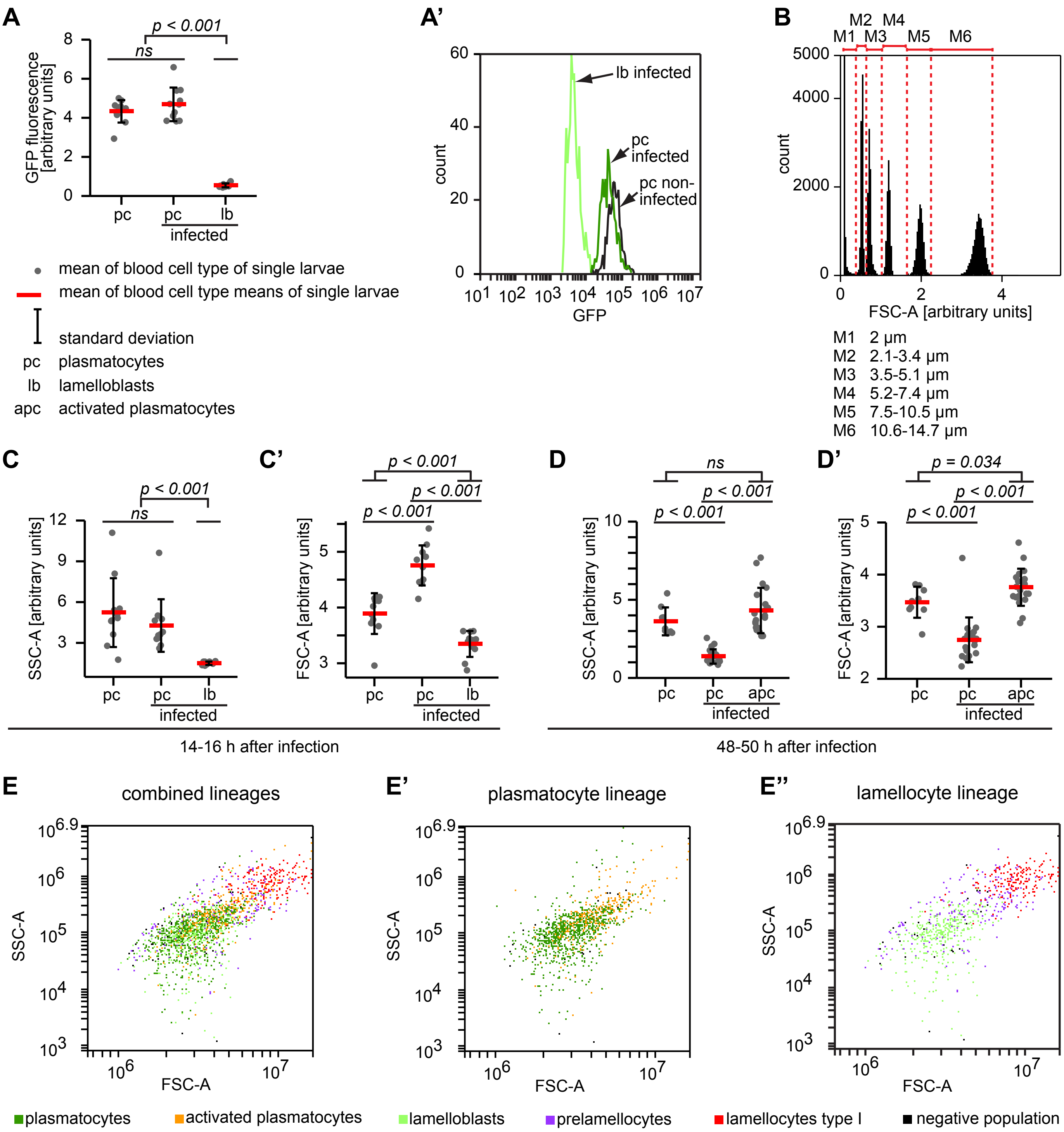


**S4 Fig. Comparison of GFP intensity, granularity, and size of plasmatocytes, lamelloblasts, and activated plasmatocytes in *L. boulardi*-infected and age-matched wild type larvae.** Values were acquired by the AccuriC6 flow cytometer. (A-A') The GFP intensity of plasmatocytes after infection is ten-fold higher than that of lamelloblasts. Fluorescent intensity values were taken 14-16 h after infection when lamelloblasts were most abundant. (B) Distribution of size beads on the FSC-A axis. (C-C') Plasmatocytes are more granular (SSC-A, C) and bigger (FSC-A, C') than lamelloblasts. Values were taken 14-16 h after infection. (D-D') After infection, plasmatocytes became less granular (SSC-A, C) and were smaller (FSC-A, C') than the plasmatocytes of uninfected and activated plasmatocytes of infected larvae. Values were taken 48-50 h after infection. (E-E'') Size and granularity of blood cells. We first gated all blood cell populations according to the gating strategy described above and then backgated the blood cell populations onto the live gate P1. Hemocyte size and granularity increased when the cells advanced in the plasmatocyte and lamellocyte lineages. A scatter plot of blood cells of all lineages (E), a scatter plot of hemocytes of the plasmatocyte lineage (E'), and a scatter plot of hemocytes of the lamellocyte lineage (E'').
