# Supplementary material for: Transdifferentiation and Proliferation in Two Distinct Hemocyte Lineages in Drosophila melanogaster Larvae after Wasp Infection
Source: PLoS Pathog. 2016 Jul 14;12(7):e1005746. doi: 10.1371/journal.ppat.1005746 (PMC4945071; doi:10.1371/journal.ppat.1005746)
Supplement: S5 Fig — (A) Total hemocyte counts of uninfected Me/w heterozygous larvae collected every second hour until 50 h. (B) Total counts after a L. boulardi, (C) a L. clavipes, and (D) a L. heterotoma infection. The box and whiskers plots depict the means of the total cell counts as red bars, the hinges of the box represent the upper and lower bound of the standard deviation (SD), and the whiskers reach to the lowest (Min) and highest (Max) measured cell number. Each dot represents the total cell count of an individual larva. In (B-D) the infection types are plotted as colored dots: Non-melanized wasp eggs as white and melanized wasp eggs as dark grey dots, living wasp larvae as light grey and killed wasp larvae as black dots. Blood cell numbers of at least ten age-matched control and L. boulardi-infected larvae were measured every second hour. The blood cells of larvae infected by L. clavipes and L. heterotoma were only counted at selected time points. Total blood cell numbers of control larvae increased slowly and rose suddenly at the two final time points (A). In L. boulardi-infected larvae, the total blood cell number suddenly increased 14 h after infection and stayed elevated (B). A similar pattern could be observed after a L. clavipes infection (C). However, total cell counts of L. heterotoma-infected larvae did not increase (D). The dynamics of total cell counts after L. boulardi and L. clavipes infections were comparatively equal, but the infection types were not. While eggs of L. clavipes started to melanize already at 22 h and were fully melanized 28 h after infection, the melanization of L. boulardi eggs was delayed. In fact, L. boulardi eggs only melanized very lightly and wasp larvae hatched around 30–32 h after infection. Wasp larvae of L. clavipes rarely hatched. The cellular immune system encapsulated the wasp eggs of L clavipes, but failed to encapsulate those of L. boulardi. Instead, L. boulardi larvae were attacked by blood cells and encapsulated. Eggs of L. het [file ppat.1005746.s005.pdf]

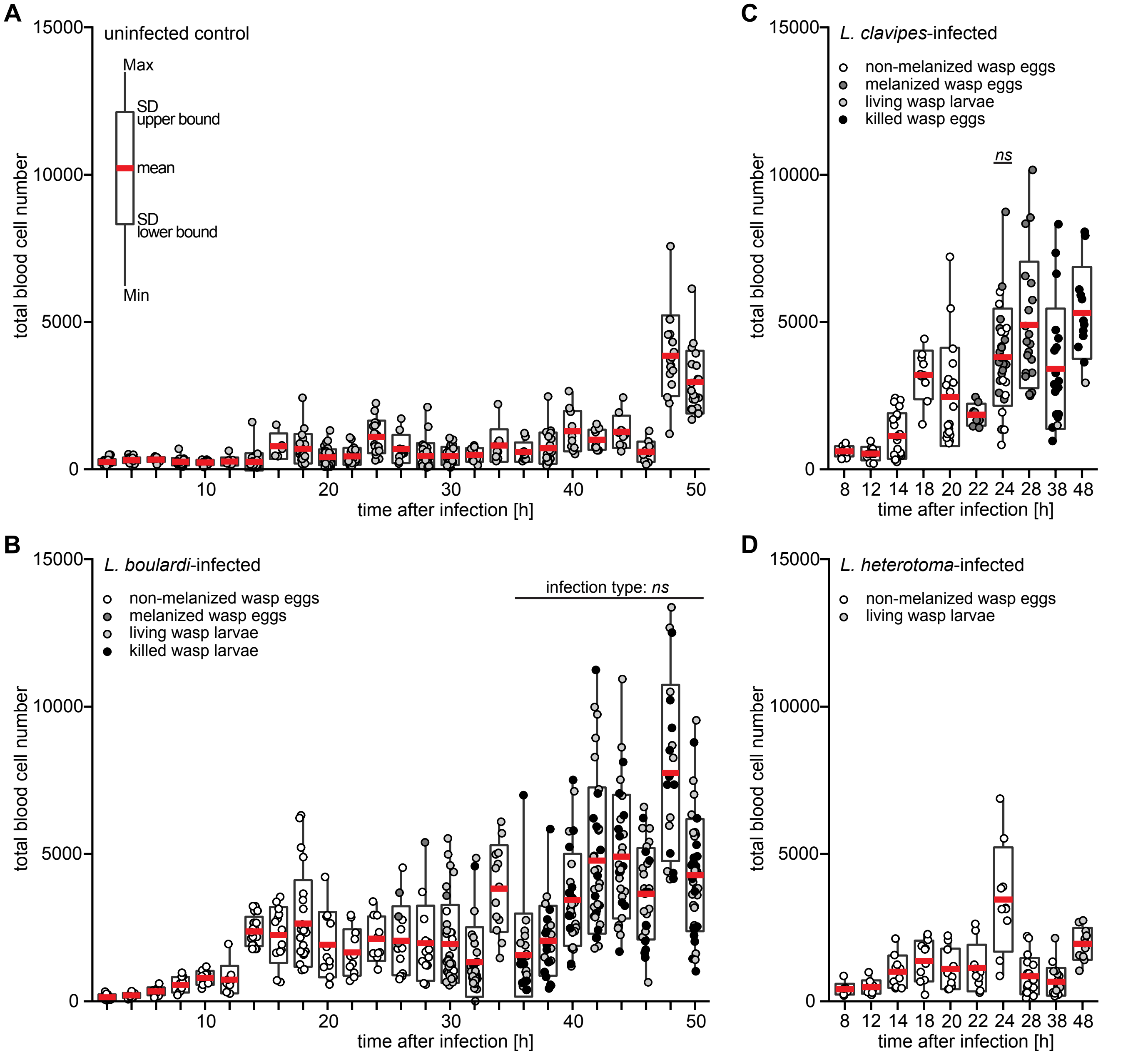

S5 Fig. Timeline of total circulating hemocyte counts of control and wasp-infected larvae. (A) Total hemocyte counts of uninfected *Me*/*w* heterozygous larvae collected every second hour until 50 h. (B) Total counts after a *L. boulardi*, (C) a *L. clavipes*, and (D) a *L. heterotoma* infection. The box and whiskers plots depict the means of the total cell counts as red bars, the hinges of the box represent the upper and lower bound of the standard deviation (SD), and the whiskers reach to the lowest (Min) and highest (Max) measured cell number. Each dot represents the total cell count of an individual larva. In (B-D) the infection types are plotted as colored dots: Non-melanized wasp eggs as white and melanized wasp eggs as dark grey dots, living wasp larvae as light grey and killed wasp larvae as black dots. Blood cell numbers of at least ten age-matched control and *L. boulardi*-infected larvae were measured every second hour. The blood cells of larvae infected by *L. clavipes* and *L. heterotoma* were only counted at selected time points. Total blood cell numbers of control larvae increased slowly and rose suddenly at the two final time points (A). In *L. boulardi*-infected larvae, the total blood cell number suddenly increased 14 h after infection and stayed elevated (B). A similar pattern could be observed after a *L. clavipes* infection (C). However, total cell counts of *L. heterotoma*-infected larvae did not increase (D). The dynamics of total cell counts after *L. boulardi* and *L. clavipes* infections were comparatively equal, but the infection types were not. While eggs of *L. clavipes* started to melanize already at 22 h and were fully melanized 28 h after infection, the melanization of *L. boulardi* eggs was delayed. In fact, *L. boulardi* eggs only melanized very lightly and wasp larvae hatched around 30-32 h after infection. Wasp larvae of *L. clavipes* rarely hatched. The cellular immune system encapsulated the wasp eggs of *L clavipes*, but failed to encapsulate those of *L. boulardi*. Instead, *L. boulardi* larvae were attacked by blood cells and encapsulated. Eggs of *L. heterotoma* were never melanized or encapsulated, nor were the wasp larvae killed. The embryonic development of *L. heterotoma* seemed to take longer than for the other two species, as 38 h after infection wasp eggs of *L. heterotoma* could still be observed in the hemocoel, whereas *L. boulardi* had started to hatch already eight hours earlier. In order to test whether blood cell numbers depended on the type of infection, we applied Wilcoxon´s rank sum test to *L. boulardi* infections starting from 36 to 50 h (B, W = 6432.5, p = 0.933, n = 230) and Welch´s Two Sample T-test to *L. clavipes* 24 h after infection (C, T = 0.815, p = 0.425, n = 20). The blood cell numbers were not dependent on infection type in either wasp species.
