# Supplementary material for: Transdifferentiation and Proliferation in Two Distinct Hemocyte Lineages in Drosophila melanogaster Larvae after Wasp Infection
Source: PLoS Pathog. 2016 Jul 14;12(7):e1005746. doi: 10.1371/journal.ppat.1005746 (PMC4945071; doi:10.1371/journal.ppat.1005746)
Supplement: S6 Fig — (A) Counts of all hemocyte classes of uninfected Me/w larvae (B-B’) and L. boulardi-infected larvae collected at the indicated time points. (C) Flow cytometry plots at three time points of Me/w and L. boulardi-infected larvae. Hemocytes of at least ten larvae were counted for each time point. The immune reactions of larvae at 25°C and 29°C were similar in the order of events. However, the immune reaction was slower due to the lower temperature. (PDF) [file ppat.1005746.s006.pdf]

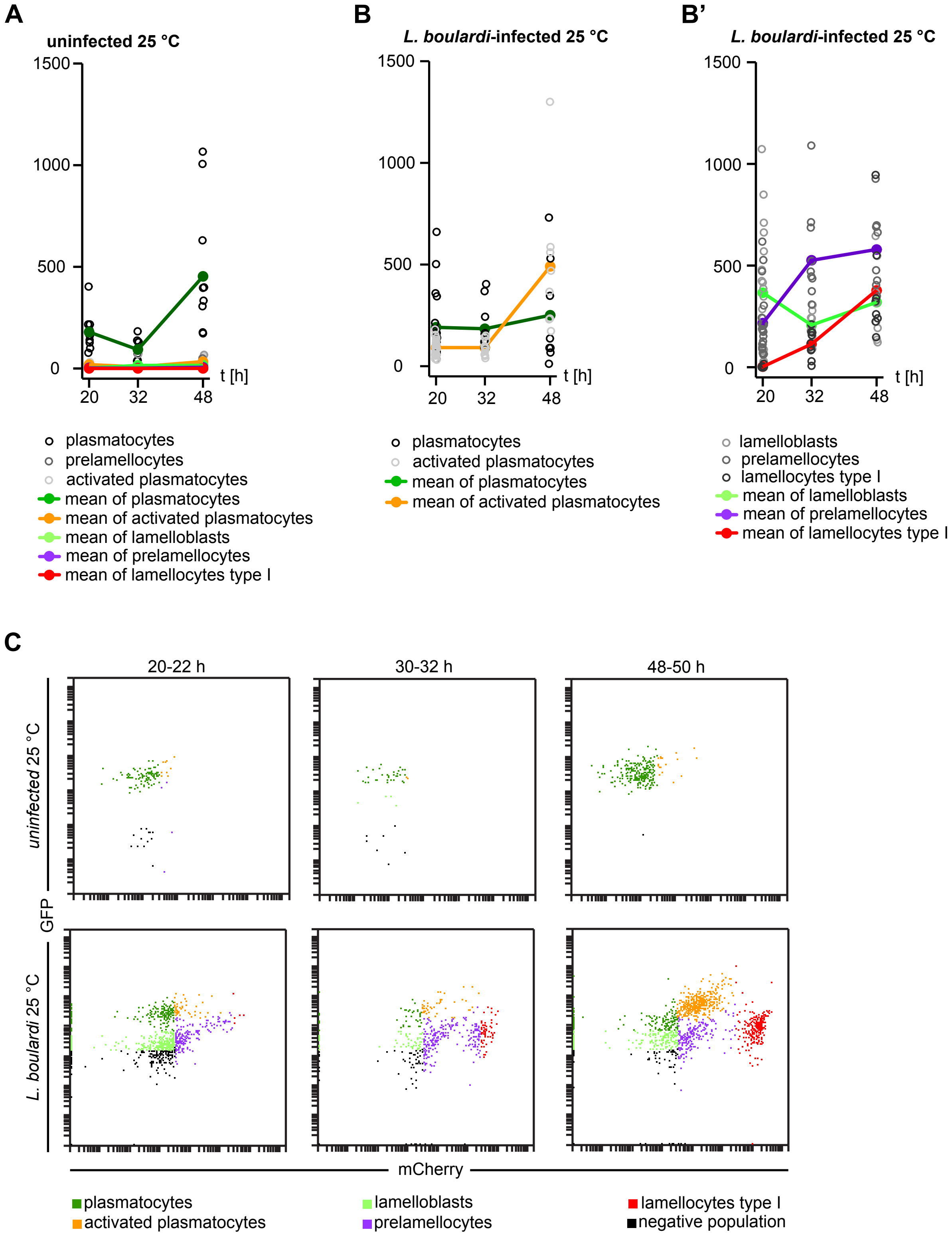

S6 Fig. Circulating hemocyte counts of three time points of age-matched control as well as *L. boulardi*-infected larvae raised at 25 °C. (A) Counts of all hemocyte classes of uninfected *Me/w* larvae (B-B') and *L. boulardi*-infected larvae collected at the indicated time points. (C) Flow cytometry plots at three time points of *Me/w* and *L. boulardi*-infected larvae. Hemocytes of at least ten larvae were counted for each time point. The immune reactions of larvae at 25 °C and 29 °C were similar in the order of events. However, the immune reaction was slower due to the lower temperature.
