# Supplementary material for: Transdifferentiation and Proliferation in Two Distinct Hemocyte Lineages in Drosophila melanogaster Larvae after Wasp Infection
Source: PLoS Pathog. 2016 Jul 14;12(7):e1005746. doi: 10.1371/journal.ppat.1005746 (PMC4945071; doi:10.1371/journal.ppat.1005746)
Supplement: S7 Fig — (A) Counts of each hemocyte class of uninfected Me/w heterozygous larvae collected at the indicated time points, (B-B’) counts of Me/w heterozygous larvae infected by L. clavipes, and (C-C’) L. heterotoma hemocytes of at least ten individual larvae were counted. All cell types of L. clavipes–infected larvae followed similar kinetics as after a L. boulardi infection. After a L. heterotoma infection however, the count of all cell types stayed low. Lamelloblasts, prelamellocytes, and lamellocytes developed, but were eradicated by the L. heterotoma venom. (PDF) [file ppat.1005746.s007.pdf]

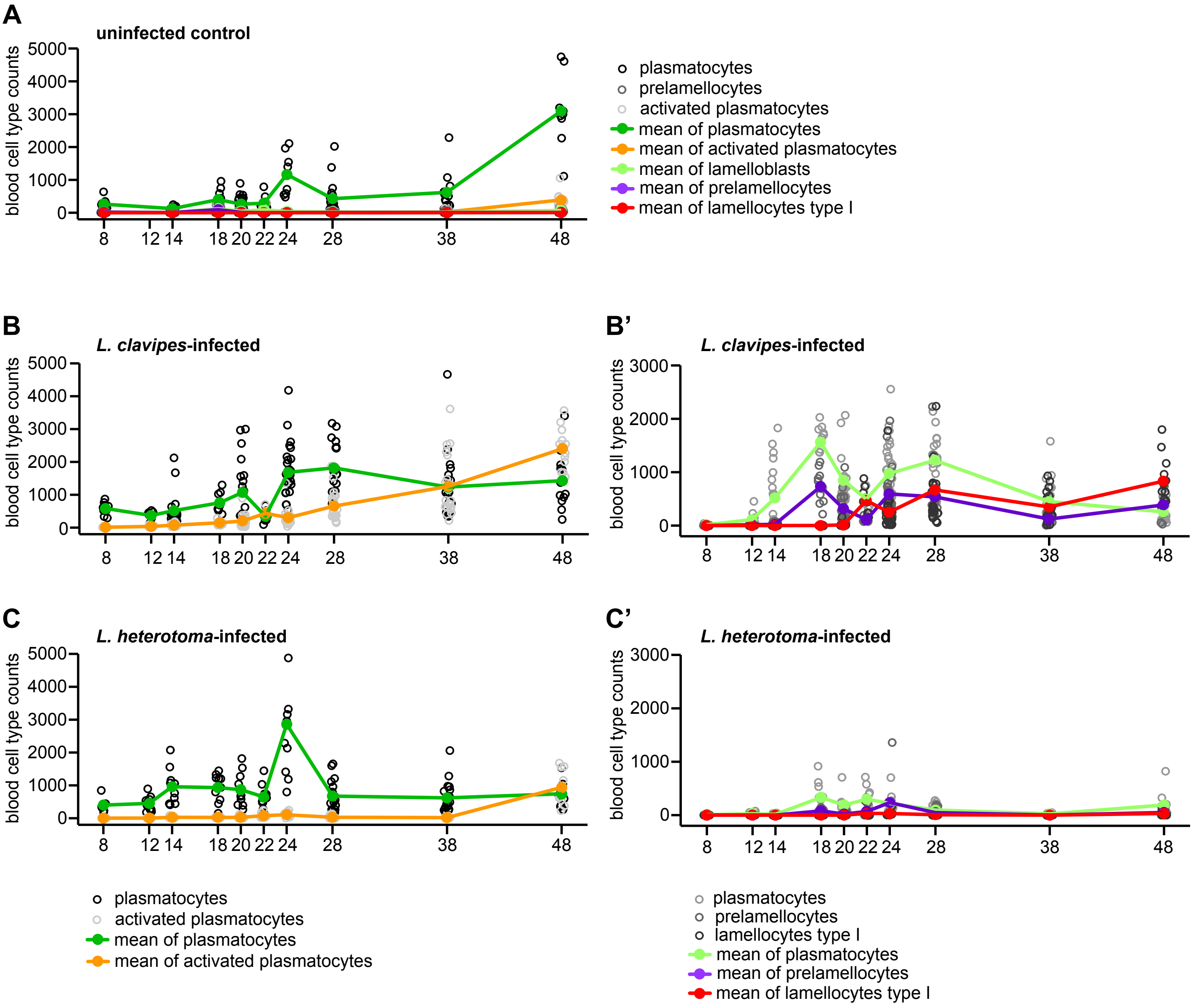

S7 Fig. Timeline of circulating hemocyte counts of age-matched control as well as *L. clavipes*- and *L. heterotoma*-infected larvae. (A) Counts of each hemocyte class of uninfected *Me*/*w* heterozygous larvae collected at the indicated time points, (B-B') counts of *Me*/*w* heterozygous larvae infected by *L. clavipes*, and (C-C') *L. heterotoma* hemocytes of at least ten individual larvae were counted. All cell types of *L. clavipes*–infected larvae followed a similar kinetics as after a *L. boulardi* infection. After a *L. heterotoma* infection however, the count of all cell types stayed low. Lamelloblasts, prelamellocytes, and lamellocytes developed, but were eradicated by the *L. heterotoma* venom.
