# Supplementary material for: Transdifferentiation and Proliferation in Two Distinct Hemocyte Lineages in Drosophila melanogaster Larvae after Wasp Infection
Source: PLoS Pathog. 2016 Jul 14;12(7):e1005746. doi: 10.1371/journal.ppat.1005746 (PMC4945071; doi:10.1371/journal.ppat.1005746)
Supplement: S8 Fig — (A-A”“) Uninfected Me/w-larvae, larvae of the same genotype infected by (B-B”“) L. boulardi, (C-C”“) L. clavipes, or (D-D”“) L. heterotoma. In uninfected larvae, plasmatocytes were the predominant blood cell type at all times. In L. boulardi- and L. clavipes-infected larvae, the first lamellocytes were seen in the circulation 18–20 h and 20–22 h after infection. At these time points, the mCherry expression was often very faint in comparison to later time points. L. heterotoma-infected larvae had only very few lamellocytes. Scale bars 50 μm. (PDF) [file ppat.1005746.s008.pdf]

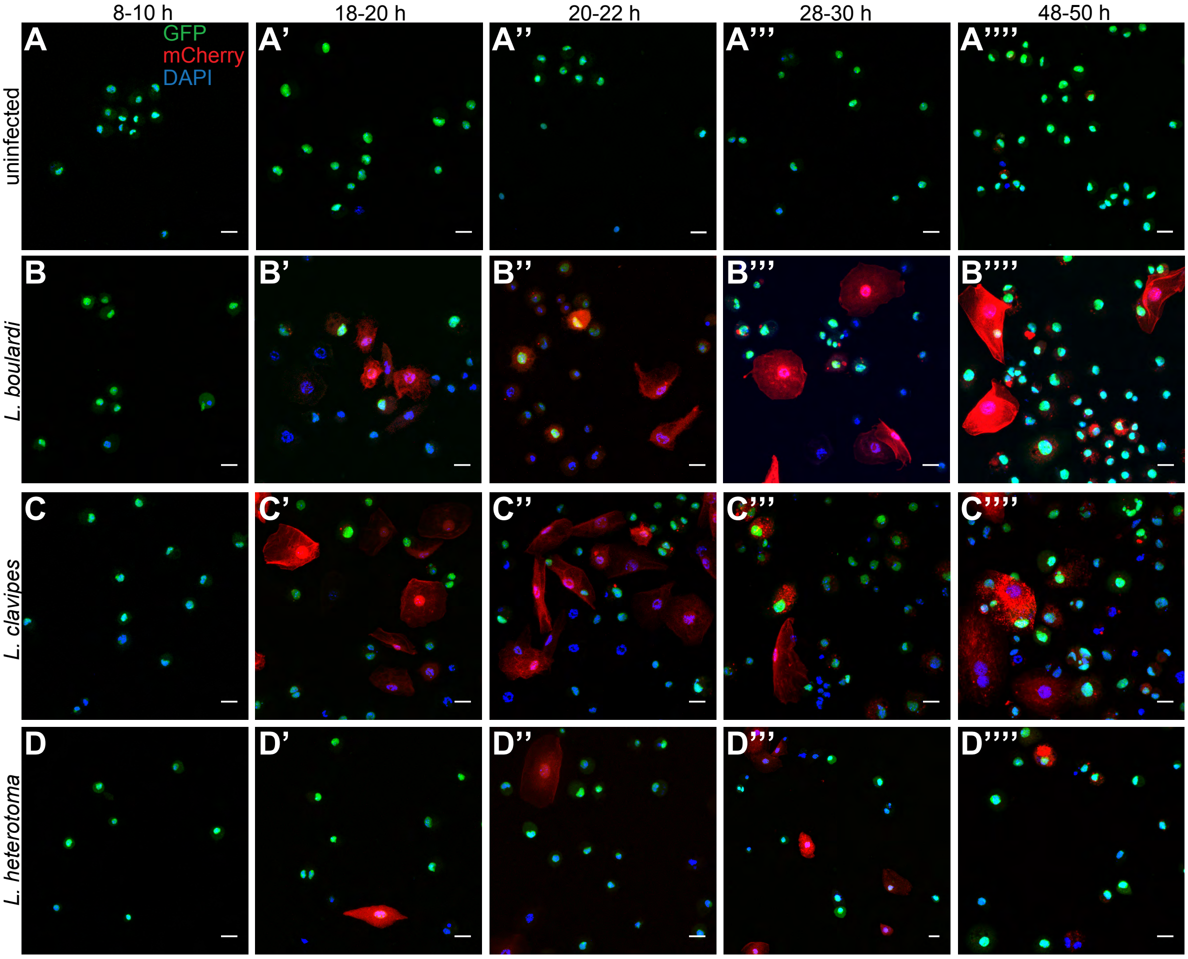

S8 Fig. Images of circulating blood cells at representative time points after wasp infection. (A-A’’’’) Uninfected *Me*/*w*-larvae, larvae of the same genotype infected by (B-B’’’’) *L. boulardi*, (C-C’’’’) *L. clavipes*, or (D-D’’’’) *L. heterotoma*. In uninfected larvae, plasmatocytes were the predominant blood cell type at all times. In *L. boulardi*- and *L. clavipes*-infected larvae, the first lamellocytes were seen in the circulation 18-20 h and 20-22 h after infection. At these time points, the mCherry expression was often very faint in comparison to later time points. *L. heterotoma*-infected larvae had only very few lamellocytes. Scale bars 50 µm.
