# Supplementary material for: Transdifferentiation and Proliferation in Two Distinct Hemocyte Lineages in Drosophila melanogaster Larvae after Wasp Infection
Source: PLoS Pathog. 2016 Jul 14;12(7):e1005746. doi: 10.1371/journal.ppat.1005746 (PMC4945071; doi:10.1371/journal.ppat.1005746)
Supplement: S9 Fig — (A) Scatter plot of hemocytes and debris. Hemocytes were easily distinguishable from debris when FSC-A was plotted against SSC-A on a logarithmic scale. However, the Click.iT Plus EdU Flow Cytometry protocol led to an additional population of non-fluorescent cells with a lower forward scatter. We assumed that these cells had died in the process of detecting dividing cells, and we pointed them out as dead cells and therefore excluded them from the analysis. All further analyses were applied to the live gate of hemocytes (red dashed ellipsoid). (B) Overlay histogram of EdU-Alexa647-stained cells with non-fluorescent blood cells (black line—non-fluorescent cells; magenta line—EdU-Alexa647-positive cells). We used blood cells of ten pooled late L3 w larvae that were previously fed with EdU and had undergone the Click-iT Plus EdU Flow Cytometry protocol with Alexa Fluor 647 picolyl azide as EdU-Alexa647-only control, and w control larvae as negative control as described earlier. EdU-Alexa647 was excited with a 640 nm diode laser and detected with FL4 equipped with a 675/25 filter. We corrected the spillover of Alexa647 by subtracting 1.61% of FL3. (C) Overlay histograms of eaterGFP/w and (D) of msnCherry/w hemocytes that had undergone the entire Click-iT Plus EdU Flow Cytometry protocol without the click reaction (green line—GFP; red line—mCherry) and normally treated hemocytes according to our protocol (black lines). The Click-iT Plus technique is an improvement of the original Click-iT reaction, where copper catalyzes the covalent reaction between an alkyne in the ethynyl moiety of EdU and a picolyl azide coupled to Alex Fluor 647. The presence of copper and reactive oxygen species damages fluorescent molecules. Adding a copper chelate reduced the toxicity for fluorescent molecules (Life technologies). However, the reaction mix still reduced the fluorescent intensities of GFP and mCherry (black arrows in C and D). We adjusted the gates accordingly. (PDF) [file ppat.1005746.s009.pdf]

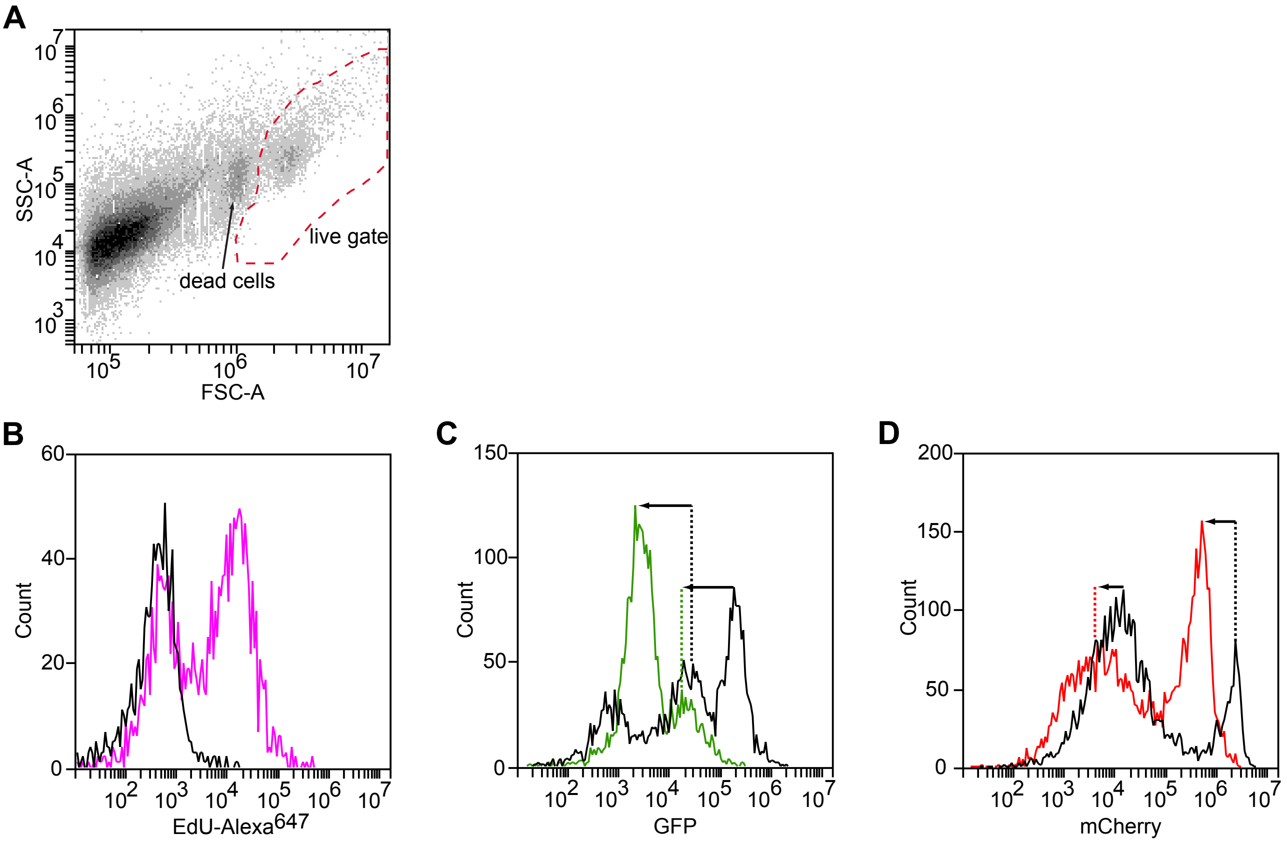

S9 Fig. Gating strategy for the dual hemocyte reporter system *Me* and EdU-Alexa^{647}-labeled hemocytes. (A) Scatter plot of hemocytes and debris. Hemocytes were easily distinguishable from debris when FSC-A was plotted against SSC-A on a logarithmic scale. However, the Click.iT® Plus EdU Flow Cytometry protocol lead to an additional population of non-fluorescent cells with a lower forward scatter. We assumed that these cells had died in the process of detecting dividing cells, and we pointed them out as dead cells and therefore excluded them from the analysis. All further analyses were applied to the live gate of hemocytes (red dashed ellipsoid). (B) Overlay histogram of EdU-Alexa^{647}-stained cells with non-fluorescent blood cells (black line – non-fluorescent cells; magenta line – EdU-Alexa^{647}-positive cells). We used blood cells of ten pooled late L3 *w* larvae that were previously fed with EdU and had undergone the Click-iT® Plus EdU Flow Cytometry protocol with Alexa Fluor® 647 picolyl azide as EdU-Alexa^{647}-only control and *w* control larvae as negative control as described earlier. EdU-Alexa^{647} was excited with a 640 nm diode laser and detected with FL4 equipped with a 675/25 filter. We corrected the spillover of Alexa^{647} by subtracting 1.61 % of FL3. (C) Overlay histograms of *eaterGFP*/*w* and (D) of *msnCherry*/*w* hemocytes that had undergone the entire Click-iT® Plus EdU Flow Cytometry protocol without the click reaction (green line – GFP; red line – mCherry) and normally treated hemocytes according to our protocol (black lines). The Click-iT® Plus technique is an improvement of the original Click-iT reaction, where copper calalyzes the covalent reaction between an alkyne in the ethynyl moiety of EdU and a picolyl azide coupled to Alex Fluor® 647. The presence of copper and reactive oxygen species damages fluorescent molecules. Adding a copper chelate reduced the toxicity for fluorescent molecules (Life technologies). However, the reaction mix still reduced the fluorescent intensities of GFP and mCherry (black arrows in C and D). We adjusted the gates accordingly.
