# Supplementary material for: Transdifferentiation and Proliferation in Two Distinct Hemocyte Lineages in Drosophila melanogaster Larvae after Wasp Infection
Source: PLoS Pathog. 2016 Jul 14;12(7):e1005746. doi: 10.1371/journal.ppat.1005746 (PMC4945071; doi:10.1371/journal.ppat.1005746)
Supplement: S10 Fig — (A-A’) Second instar Me/w larvae were infected by L. boulardi for two hours and subsequently placed on EdU-containing fly food for 12 h after a wasp infection. Hemocytes of these larvae were collected and stained immediately after feeding. The majority of hemocytes of infected larvae had incorporated EdU, indicating that they had been dividing. Hemocytes from control larvae had also divided at this time point. Stars in panel A’ mark an example of a lamelloblast and a plasmatocyte that incorporated EdU. (B-B’) EdU was fed to larvae 4–8 h after a wasp infection. Larvae were placed back on normal food and then dissected 28 h after infection. At this time point, the majority of prelamellocytes and lamellocytes type I were EdU-positive, suggesting that they originated from cells that had divided early after the infection. (C-C’) Larvae were feeding on normal fly food until 28 h after infection, after which they were put on EdU-food for four hours. Then, they were returned to normal food until they were dissected 48 h after infection. Then, only a small proportion of lamellocytes were EdU-positive, whereas other hemocyte types were EdU-positive at varying proportions, indicating that type I lamellocytes themselves were not dividing but all other cell types were. In B’ and C’ EdU-positive cells are marked with a star, examples of lamellocytes are marked with an arrow, activated plasmatocytes with filled arrowheads and prelamellocytes with open arrowheads. (PDF) [file ppat.1005746.s010.pdf]

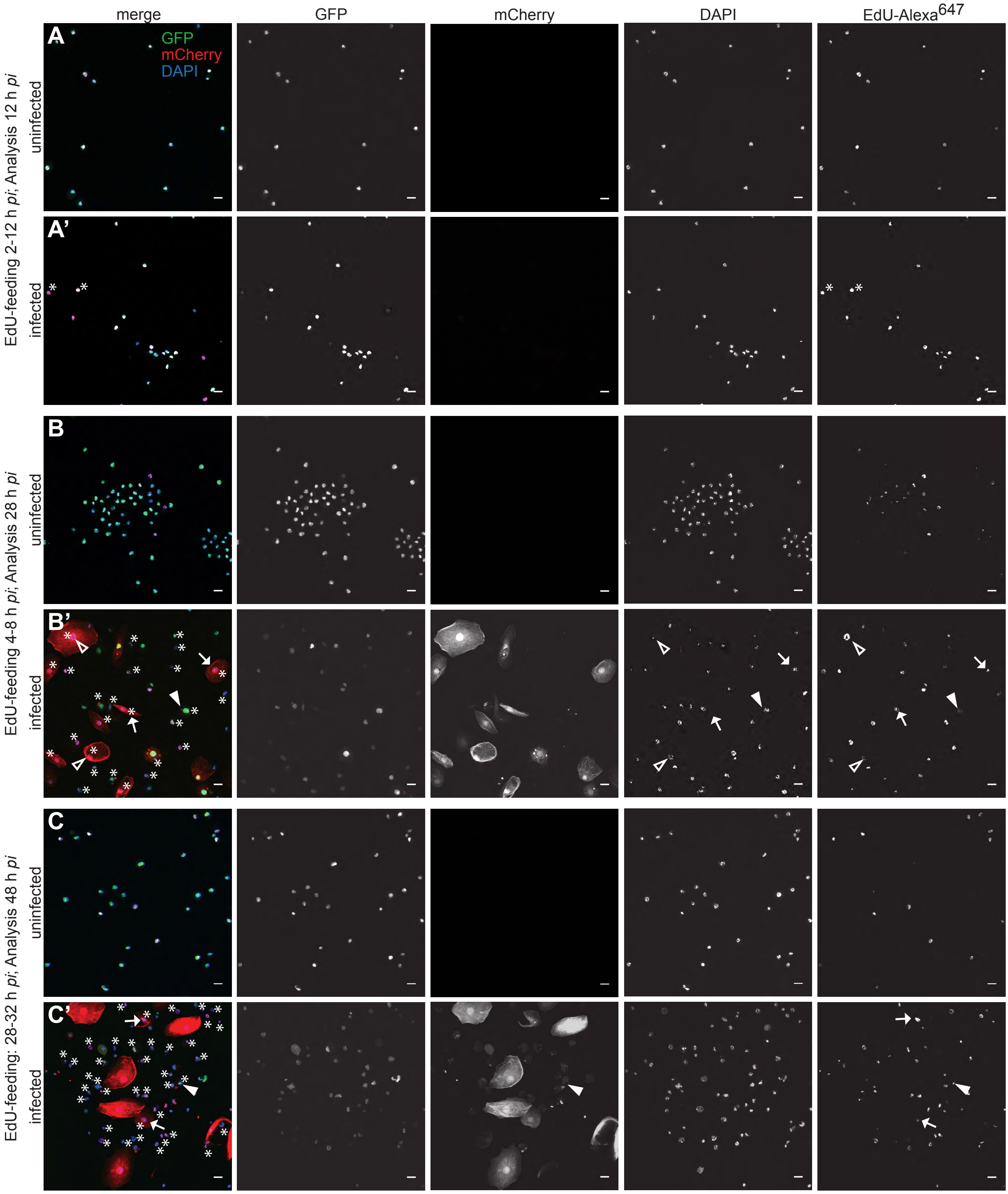

S10 Fig. ***In vivo*** **EdU incorporation of hemocytes after a wasp infection.** (A-A') Second instar *Me/w* larvae were infected by *L. boulardi* for two hours and subsequently placed on EdU-containing fly food for 12 h after a wasp infection. Hemocytes of these larvae were collected and stained immediately after feeding. The majority of hemocytes of infected larvae had incorporated EdU, indicating that they had been dividing. Hemocytes from control larvae had also divided at this time point. Stars in panel A' mark an example of a lamelloblast and a plasmatocyte that incorporated EdU. (B-B') EdU was fed to larvae 4-8 h after a wasp infection. Larvae were placed back on normal food and then dissected 28 h after infection. At this time point, the majority of prelamellocytes and lamellocytes type I were EdU-positive, suggesting that they originated from cells that had divided early after the infection. (C-C') Larvae were feeding on normal fly food until 28 h after infection, after which they were put on EdU-food for four hours. Then, they were returned to normal food until they were dissected 48 h after infection. Then, only a small proportion of lamellocytes were EdU-positive, whereas other hemocyte types were EdU-positive at varying proportions, indicating that type I lamellocytes themselves were not dividing but all other cell types were. In B' and C' EdU-positive cells are marked with a star, examples of lamellocytes are marked with an arrow, activated plasmatocytes with filled arrowheads and prelamellocytes with open arrowheads.
