# Supplementary material for: Transdifferentiation and Proliferation in Two Distinct Hemocyte Lineages in Drosophila melanogaster Larvae after Wasp Infection
Source: PLoS Pathog. 2016 Jul 14;12(7):e1005746. doi: 10.1371/journal.ppat.1005746 (PMC4945071; doi:10.1371/journal.ppat.1005746)
Supplement: S11 Fig — (A-C) Quantification of different EdU experiments explained in the S10 Fig caption. (PDF) [file ppat.1005746.s011.pdf]

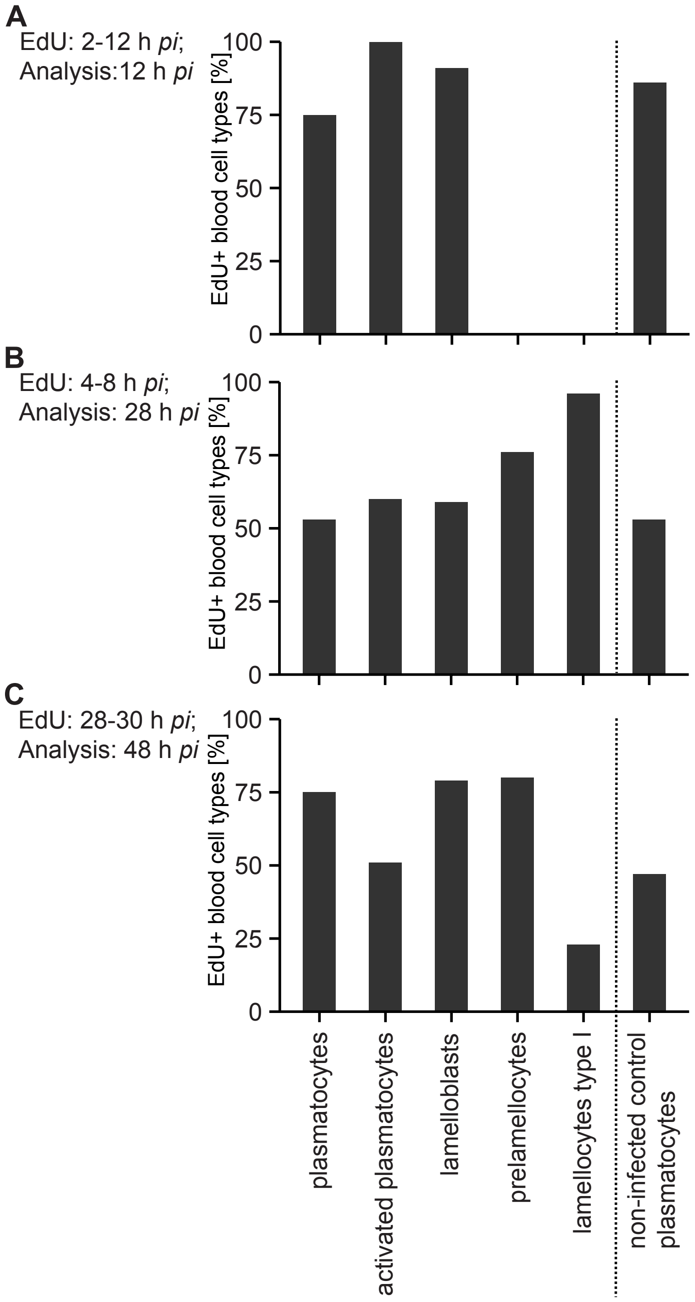

S11 Fig. Quantification of EdU results. (A-C) Quantification of different EdU experiments explained in the S10 Fig caption.
