# Supplementary material for: Transdifferentiation and Proliferation in Two Distinct Hemocyte Lineages in Drosophila melanogaster Larvae after Wasp Infection
Source: PLoS Pathog. 2016 Jul 14;12(7):e1005746. doi: 10.1371/journal.ppat.1005746 (PMC4945071; doi:10.1371/journal.ppat.1005746)
Supplement: S12 Fig — Second instar Me/w larvae were infected by L. boulardi for 2 h, hemocytes were collected at 8, 18, 20, 28, and 48 h after infection and stained with NimC1/P1 (first two panels), L4 (two middle panels) and L6 (two last panels) antibodies. For clarity, NimC1/P1, L4 and L6 stains are shown as layers separated from respective merged image. Hemocytes expressing GFP (plasmatocytes and lamelloblasts, activated plasmatocytes and prelamellocytes) were NimC1 positive after infection (A’-E’). Mature lamellocytes with no GFP expression were always NimC1 negative (B’-E’). Some, but not all, of the activated plasmatocytes were also stained with L4 (filled arrowheads, G’) as were prelamellocytes (G’) and mature lamellocytes (H’-J’). Some of the plasmatocytes were L4-postive already 8 hours after a wasp infection (F’), indicating their activation and/or transformation into lamellocytes. L6-positive hemocytes were detected at 18 h after infection (K-O). L6 mainly stained discoidal prelamellocytes (L-L’) and lamellocytes (L-O), but we also detected some L6-positive activated lamellocytes (N-N’). Arrows point to examples of lamellocytes, filled arrowheads to activated plasmatocytes and open arrowheads to prelamellocytes. “Greater than” signs (>) in M and M’ point to a lamellocyte type II that was stained with L6. Stars in D and D’ show a GFP and mCherry-negative, elongated cell that was stained with NimC1/P1 and in L and L’ a cluster of mCherry negative lamellocytes that were stained with L6. Size bars 10 μm. (PDF) [file ppat.1005746.s012.pdf]

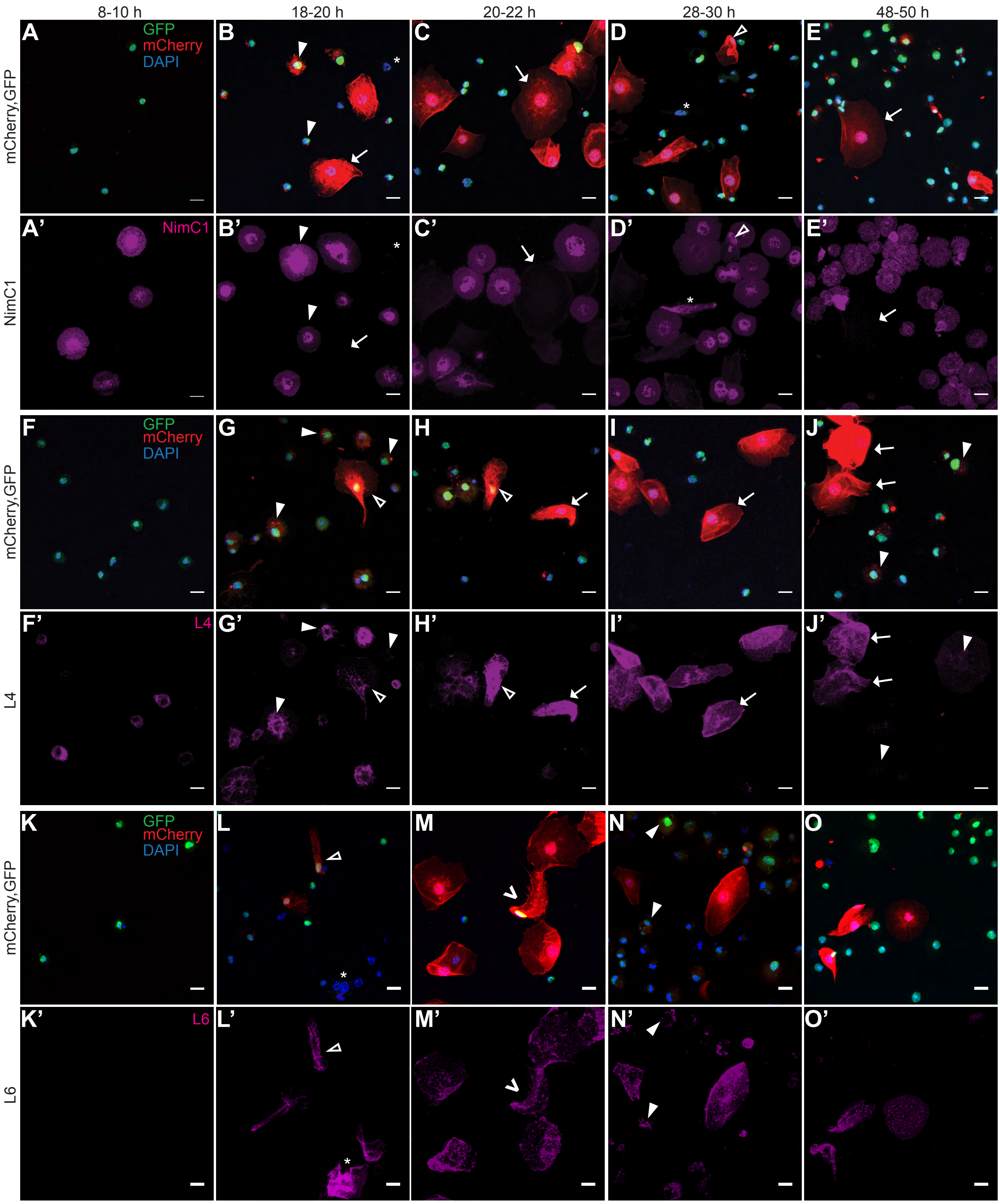

S12 Fig. Visualization of hemocyte types with NimC1/P1, L4, and L6 hemocyte antibodies after a wasp infection. Second instar *Me*/*w* larvae were infected by *L. boulardi* for 2 h, hemocytes were collected at 8, 18, 20, 28, and 48 h after infection and stained with NimC1/P1 (first two panels), L4 (two middle panels) and L6 (two last panels) antibodies. For clarity, NimC1/P1, L4 and L6 stains are shown as layers separated from respective merged image. Hemocytes expressing GFP (plasmatocytes and lamelloblasts, activated plasmatocytes and prelamellocytes) were NimC1 positive after infection (A'-E'). Mature lamellocytes with no GFP expression were always NimC1 negative (B'-E'). Some, but not all, of the activated plasmatocytes were also stained with L4 (filled arrowheads, G') as were prelamellocytes (G') and mature lamellocytes (H'-J'). Some of the plasmatocytes were L4-postive already 8 hours after a wasp infection (F'), indicating their activation and/or transformation into lamellocytes. L6-positive hemocytes were detected at 18 h after infection (K-O). L6 mainly stained discoidal prelamellocytes (L-L') and lamellocytes (L-O), but we also detected some L6-positive activated lamellocytes (N-N'). Arrows point to examples of lamellocytes, filled arrowheads to activated plasmatocytes and open arrowheads to prelamellocytes. "Greater than" signs (>) in M and M' point to a lamellocyte type II that was stained with L6. Stars in D and D' show a GFP and mCherry-negative, elongated cell that was stained with NimC1/P1 and in L and L' a cluster of mCherry negative lamellocytes that were stained with L6. Size bars 10 µm.
