# Supplementary material for: Transdifferentiation and Proliferation in Two Distinct Hemocyte Lineages in Drosophila melanogaster Larvae after Wasp Infection
Source: PLoS Pathog. 2016 Jul 14;12(7):e1005746. doi: 10.1371/journal.ppat.1005746 (PMC4945071; doi:10.1371/journal.ppat.1005746)
Supplement: S1 Table — (PDF) [file ppat.1005746.s013.pdf]

S1 Table. Wasp species used in the study.

**Wasp species**	**Strain**	**Source/Reference**
Leptopilina boulardi	*LbG486*	Yves Carton via Istvan Ando
Leptopilina clavipes	*LcNet*	Todd Schlenke
Leptopilina heterotoma	*Lh14*	Todd Schlenke
