# Supplementary material for: Transdifferentiation and Proliferation in Two Distinct Hemocyte Lineages in Drosophila melanogaster Larvae after Wasp Infection
Source: PLoS Pathog. 2016 Jul 14;12(7):e1005746. doi: 10.1371/journal.ppat.1005746 (PMC4945071; doi:10.1371/journal.ppat.1005746)
Supplement: S2 Table — (PDF) [file ppat.1005746.s014.pdf]

**S2 Table. Fly stocks used in the study.**

| Genotype | Abbreviation | Phenotype | Citation/ Source |
|---|---|---|---|
| *w*$^{1118}$ *iso* | *w* | | [1] |
| *eater-GFP* | *eaterGFP* | GFP | [2] |
| *MSNF9MO-mCherry* | *msnCherry* | mCherry | [3] |
| *msnCherry,eaterGFP* | *Me* | mCherry, GFP | |
| *eater-DsRed* | *eaterDsRed* | DsRed | [2] |
| *Hml*$^{\Delta}$*-GAL4* | *Hml*$^{\Delta}$*>* | | [4] |
| *msnCherry;eaterDsRed,Hml*$^{\Delta}$*>* | | mCherry, DsRed | |
| *UAS-S/G2/M-Green* | *S/G2/M-Green* | Green | [5] |
| *He-GAL4* | *He>* | | [6] |
| *UAS-edin* | *edin* | | [7] |
| *UAS-edin*$^{KK109528}$ | *edin*$^{KK}$ | | VDRC |
| *Me;edin*$^{KK}$ | | | [8] |
| *Me;Fb-GAL4* | *Me;Fb>* | | [8] |

**References for S2 Table**


1. Ryder E, Blows F, Ashburner M, Bautista-Llacer R, Coulson D, Drummond J, et al. The DrosDel collection: a set of P-element insertions for generating custom chromosomal aberrations in Drosophila melanogaster. Genetics. 2004; 167: 797-813. doi: 10.1534/genetics.104.026658

2. Kroeger PT, Jr., Tokusumi T, Schulz RA. Transcriptional regulation of *eater* gene expression in *Drosophila* blood cells. Genesis. 2012; 50: 41-49. doi: 10.1002/dvg.20787

3. Tokusumi T, Sorrentino RP, Russell M, Ferrarese R, Govind S, Schulz RA. Characterization of a lamellocyte transcriptional enhancer located within the *misshapen* gene of *Drosophila melanogaster*. PLoS One. 2009; 4: e6429. doi: 10.1371/journal.pone.0006429

4. Sinenko SA, Mathey-Prevot B. Increased expression of *Drosophila* tetraspanin, Tsp68C, suppresses the abnormal proliferation of ytr-deficient and Ras/Raf-activated hemocytes. Oncogene. 2004; 23: 9120-9128. doi: 10.1038/sj.onc.1208156

5. Nakajima Y, Kuranaga E, Sugimura K, Miyawaki A, Miura M. Nonautonomous apoptosis is triggered by local cell cycle progression during epithelial replacement in *Drosophila*. Mol Cell Biol. 2011; 31: 2499-2512. doi: 10.1128/MCB.01046-10

6. Zettervall CJ, Anderl I, Williams MJ, Palmer R, Kurucz E, Ando I, et al. A directed screen for genes involved in *Drosophila* blood cell activation. Proc Natl Acad Sci USA. 2004; 101: 14192-14197. doi: 10.1073/pnas.0403789101

7. Vanha-aho LM, Kleino A, Kaustio M, Ulvila J, Wilke B, Hultmark D, et al. Functional characterization of the infection-inducible peptide Edin in Drosophila melanogaster. PLoS One. 2012; 7: e37153. doi: 10.1371/journal.pone.0037153

8. Vanha-aho LM, Anderl I, Vesala L, Hultmark D, Valanne S, Rämet M. *Edin* expression in the fat body is required in the defense against parasitic wasps in *Drosophila melanogaster*. PLoS Pathog. 2015; 11: e1004895. doi: 10.1371/journal.ppat.1004895
